# Quantifying Adaptive Evolution in the *Drosophila* Immune System

**DOI:** 10.1371/journal.pgen.1000698

**Published:** 2009-10-23

**Authors:** Darren J. Obbard, John J. Welch, Kang-Wook Kim, Francis M. Jiggins

**Affiliations:** 1 Institute of Evolutionary Biology, University of Edinburgh, Edinburgh, United Kingdom; 2 Department of Animal and Plant Sciences, University of Sheffield, Sheffield, United Kingdom; 3 Department of Genetics, University of Cambridge, Cambridge, United Kingdom; University of California Davis, United States of America

## Abstract

It is estimated that a large proportion of amino acid substitutions in *Drosophila* have been fixed by natural selection, and as organisms are faced with an ever-changing array of pathogens and parasites to which they must adapt, we have investigated the role of parasite-mediated selection as a likely cause. To quantify the effect, and to identify which genes and pathways are most likely to be involved in the host–parasite arms race, we have re-sequenced population samples of 136 immunity and 287 position-matched non-immunity genes in two species of *Drosophila*. Using these data, and a new extension of the McDonald-Kreitman approach, we estimate that natural selection fixes advantageous amino acid changes in immunity genes at nearly double the rate of other genes. We find the rate of adaptive evolution in immunity genes is also more variable than other genes, with a small subset of immune genes evolving under intense selection. These genes, which are likely to represent hotspots of host–parasite coevolution, tend to share similar functions or belong to the same pathways, such as the antiviral RNAi pathway and the IMD signalling pathway. These patterns appear to be general features of immune system evolution in both species, as rates of adaptive evolution are correlated between the *D. melanogaster* and *D. simulans* lineages. In summary, our data provide quantitative estimates of the elevated rate of adaptive evolution in immune system genes relative to the rest of the genome, and they suggest that adaptation to parasites is an important force driving molecular evolution.

## Author Summary

All organisms are attacked by an ever-changing array of pathogens and parasites, and it is widely supposed that the ensuing host–parasite “arms race” must drive extensive adaptive evolution in genes of the immune system. Here we have taken advantage of new sequencing technologies and analytical approaches to quantify the amount of adaptation that is occurring in immunity genes relative to the rest of the genome. We sampled two species of fruit fly (*D. melanogaster* and *D. simulans*) from eight different populations around the world, and sequenced 136 immunity and 287 non-immunity genes from these samples. Based on the differences in the sequences between the two species, and the genetic diversity within each species, we have estimated that natural selection drives twice as much change in immune-related proteins as in proteins with no immune function. Interestingly, the rate of adaptation is also more variable among immunity genes than among other genes in the genome, with a small subset of immunity genes evolving under intense natural selection. We suggest that these genes may represent hotspots of host–parasite coevolution within the genome.

## Introduction

Hosts face an ever-changing array of parasites to which they must adapt, and parasites are widely believed to be one of the most important and universal selection pressures in natural populations. Consistent with this view, immune genes in several taxa are known to evolve faster than other genes, and sometimes significantly faster than the neutral rate – a signature of adaptive evolution [1],[2],[3]. Indeed, many studies of one or a few immune genes have identified the action of positive selection in *Drosophila*, including Relish [4], the Scavenger Receptors [5] RNAi genes [6], TEPs [7], Persephone [8] and others [2]. More recently, complete genome sequencing of multiple *Drosophila* species found that immune-related genes have high rates of amino-acid substitution, and are more likely to show evidence of adaptive evolution than other genes [1],[9]. Here we go beyond the yes/no detection of selection, to quantify the additional adaptation that occurs in proteins of the immune system over and above that which occurs in the rest of the genome.

The rate at which natural selection fixes new mutations can be estimated by comparing the amount of polymorphism within populations to divergence between species at synonymous and nonsynonymous sites [10],[11],[12],[13],[14]. Approaches of this kind have been used to estimate the genome-wide rate of adaptive evolution, and found that it is often surprisingly high [10],[13],[15],[16],[17]. However, the nature of the selection pressures underlying this evolution remains unknown.

One approach to answering this question is to compare estimated rates of adaptive evolution between proteins with different functions. Moreover, focussing on genes where we have a strong expectation of elevated positive selection also has a further benefit; there is an ongoing debate about the extent to which the high genomic estimates represent artefacts of processes such as population demography [15],[18],[19], and testing the *a priori* hypothesis that immunity genes will have increased adaptive rates can address this issue.

**Figure 1:**
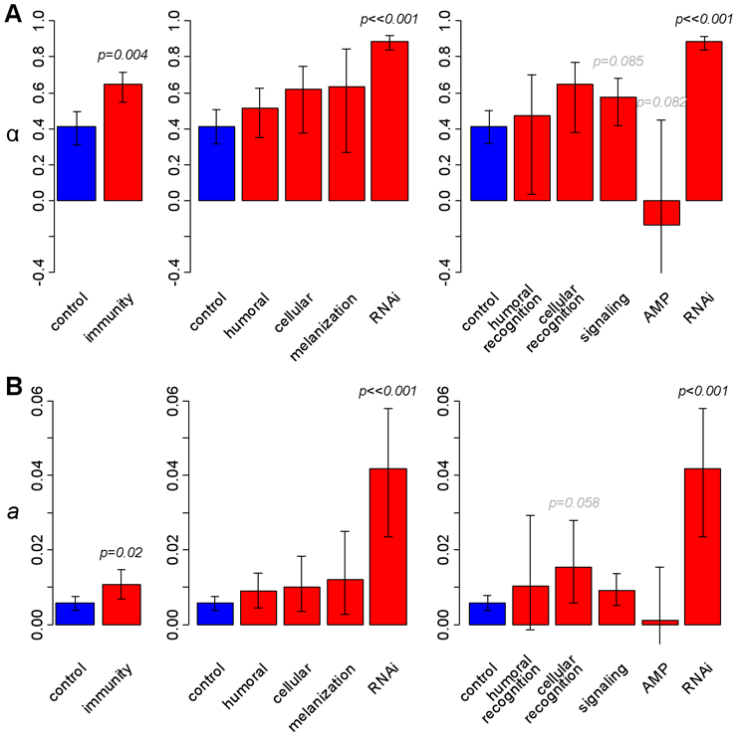
The estimated rate of adaptive substitution in different classes of gene. Estimates from a single Kenyan population sample from each of *D. melanogaster* and *D. simulans*, and the divergence between them. (A) estimates of the proportion of non-synonymous substitutions that were adaptive (α). (B) estimates of the number of adaptive non-synonymous substitutions per non-synonymous site (*a*). P-values are with respect to the control genes, and were determined by bootstrapping. Error bars are 95% bootstrap intervals around the mean, calculated across loci.

**Figure 2:**
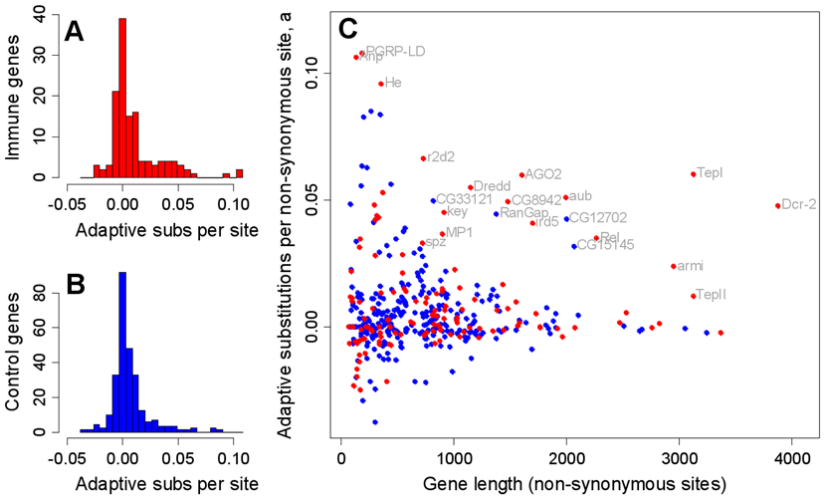
Immune genes have a greater variance than other genes in the estimated rate of adaptive substitution. The estimated number of adaptive substitutions per non-synonymous site between *D. melanogaster* and *D. simulans*, for 131 immune genes (A; red) and 265 control genes (B; blue). The mean and variance is higher for immune genes than control genes: 0.011 *vs.* 0.006 (*p* = 0.022) and 0.00054 *vs.* 0.00026 (*p* = 0.018) respectively, though the modes are extremely similar (the modal class in both (B) and (C) is centred on zero [−0.003,0.003]). (C) shows number of adaptive substitutions per non-synonymous site, plotted against gene length. We used *a* in place of α for this analysis because α is poorly estimated for single genes (see Materials and Methods).

**Figure 3:**
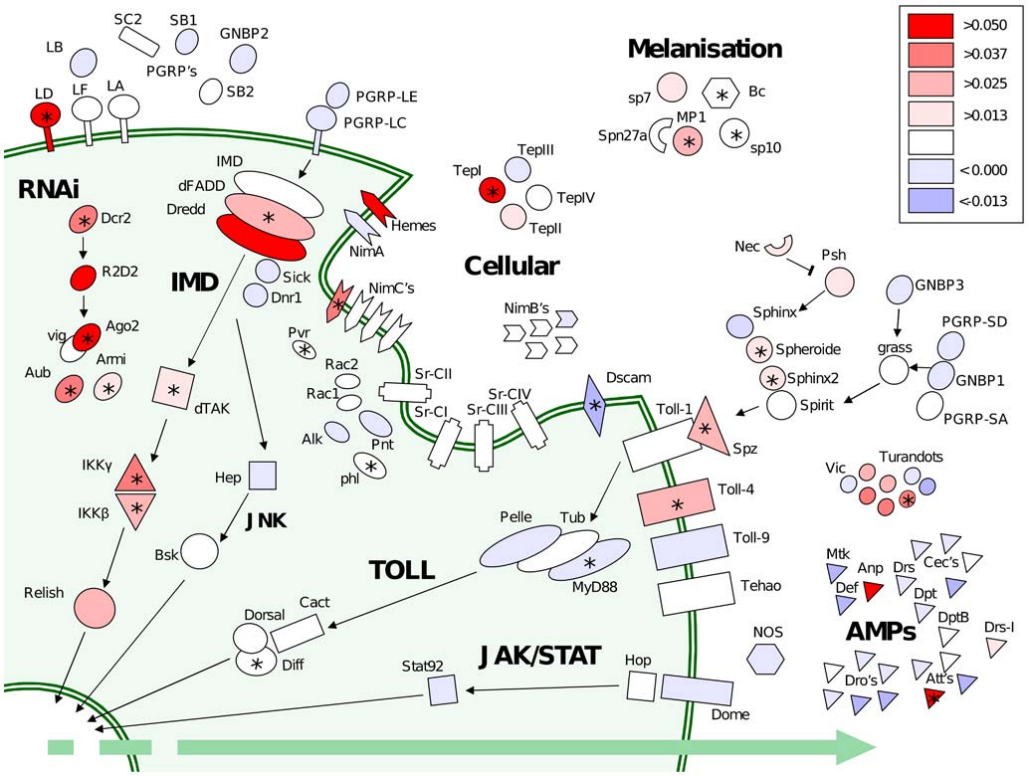
Immunity pathways and genes coloured according to their estimated rate of adaptive evolution. Well-characterised immune-related genes arranged by pathway and cellular location, coloured according to the inferred rate of adaptive substitution (*a*: adaptive substitutions per non-synonymous site between *D. melanogaster* and *D. simulans*). Red indicates high rates of adaptive substitution, blue indicates an excess of weakly-deleterious polymorphism. Asterisks indicate those genes that individually display a significant deviation from neutrality in a classical single-locus MK test using the data presented here. In addition to effect size, single-gene significance also strongly reflects the power of the test and will be affected by (e.g.) gene length. To achieve maximum coverage of the immune system, the analysis presented in this figure uses all the sampled populations of *D. melanogaster* and *D. simulans*.

**Figure 4:**
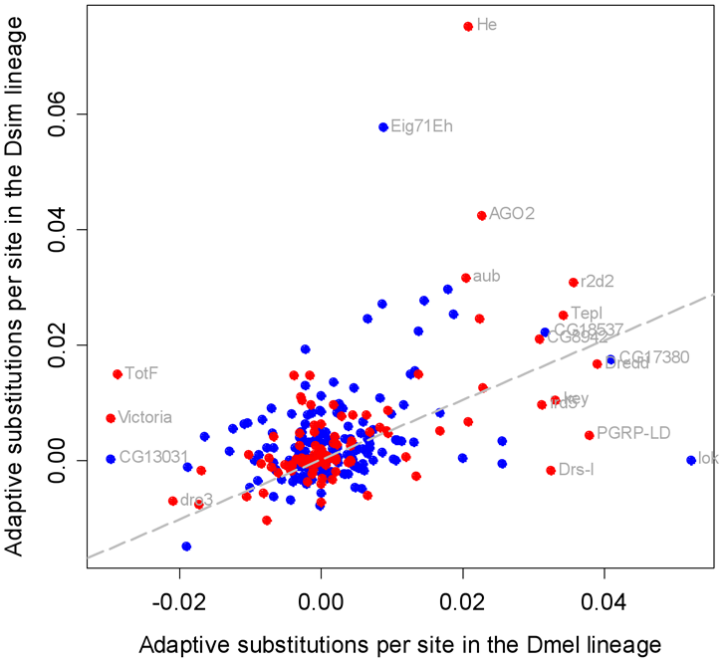
The estimated rate of adaptive substitution is correlated between the *D. melanogaster* and *D. simulans* lineages. The estimated number of adaptive substitutions per non-synonymous site, *a*, estimated independently along the *D. melanogaster* and *D. simulans* lineages (immune genes in red, control genes in blue). Spearman's rank correlation coefficient is significantly positive, indicating that genes with high rates of adaptive substitution in one lineage tend also to have high rates in the other (ρ = 0.36, *p* = 2×10^−10^). The correlation does not differ between immune genes and other genes (ρ = 0.47 *vs.* 0.29, *p* = 0.14 by bootstrapping), and the result is the same when using all populations (ρ = 0.51 *vs.* 0.35, *p* = 0.16).

To assess the role of pathogens and other parasites as a cause of molecular evolution we have resequenced population samples of most of the best-characterised immunity genes in the *Drosophila melanogaster* genome, together with position-matched ‘control’ genes with no known immune function. This provides a quantitative estimate of the impact of parasite-mediated selection on the rate of adaptive evolution, and suggests that immunity genes have double the genome-average rate (Figure 1). We found that this was not caused by a generally elevated rate in immunity genes. Instead, most immunity genes show similar rates of adaptive evolution to the rest of the genome, with only a small subset evolving under very intense selection (Figure 2). These genes tend to be concentrated in a few pathways, which we argue are likely to be hotspots of host-parasite coevolution (Figure 3). Interestingly, these pathways are known to be suppressed by pathogens, and this suggests that active parasite-suppression of the immune system is an important cause of this adaptive evolution. Furthermore, when independent lineages are compared, similar genes show accelerated rates of adaptation (Figure 4). This suggests that despite their dynamic nature, host-parasite interactions may create similar selective pressures in related species, leading to replicable signatures at the molecular level.

## Results

We have resequenced 136 of the best characterised immunity genes in *Drosophila melanogaster* and *D. simulans*. To get an unbiased estimate of the background rate of adaptive evolution, we also sampled position-matched ‘control’ genes with no known immune function. We sampled flies from six *D. melanogaster* populations and two *D. simulans* populations, and pooled genomic DNA from four outcrossed flies (eight alleles of each gene) from each population. We then amplified the target genes by PCR, and sequenced them using the Solexa-Illumina platform. After excluding sites with less than 20-fold coverage (Figure S1) and genes represented by less than 100 bp of sequence, there remained a total of 462.7 kbp of protein coding sequence from *D. melanogaster* representing 415 genes, and 335.6 kbp from *D. simulans* representing 309 genes. In this coding sequence we identified 12,974 putative SNPs in *D. melanogaster* and 10,759 in *D. simulans*. Raw data are available from the NCBI Short Read Archive under accession number SRA009020, or on request from the authors, and data for individual genes is given in Table S1.

Short-read sequencing of long PCR products provides a cost-efficient approach to identifying polymorphic sites and to estimating levels of genetic diversity, and has been shown to be as, or more, accurate than traditional Sanger sequencing [20]. By pooling template DNA between multiple individuals, cost-efficiency can be improved even further, though this may come at the cost of reduced accuracy. To assess the quality of our pooled-template short-read data, we re-sequenced 11 loci in two populations from diploid genomic DNA of the same individuals, using traditional Sanger sequencing (a total of 12,415 bp; see Text S1 and Figures S2, S3, S4, S5, S6, S7, S8, S9 for a detailed analysis of data quality and a comparison of the methods). We found that our pooled-template short-read approach successfully recovered ∼90% of the polymorphisms identified by Sanger sequencing, and more than 94% of short-read polymorphisms were verified by the Sanger data. Assuming the Sanger sequences are correct, on a per-site basis, this is an accuracy of 99.8%. Although estimates of allele-frequency are relatively poor (the correlation between Sanger and short-read estimates was Pearson's ρ = 0.71), our estimates of genetic diversity are highly correlated between the two methods (Pearson's ρ = 0.94 and 0.90 for per locus estimates of θ_w_ and θ_π_ respectively). Our approach compares favourably with automated Sanger-sequencing of diploid genomic DNA, which is reported to have an error rate of ∼7% of SNPs [reviewed in 20]. However, as with related methods [21], the majority of our sequencing errors appear to result from PCR (allelic dropout and misincorporation of bases) or unequal mixing of template DNA. Because of this, future mixed-template studies may be improved by the use of direct DNA-capture in place of PCR, and/or mixing larger numbers of individuals, so that read-frequency better-reflects population allele-frequency.

For the following analyses of adaptive rates we focus on Kenyan populations of each species, as these are thought to be representative their ancestral range [22], and should minimise demographic artefacts associated with recent colonisation [14],[15],[18]. However, analyses of combined data, which give very similar results, are presented in Figures S10, S11, S12, S13, S14, S15.

### Immunity genes show higher rates of adaptive evolution than other genes

The proportion of amino acid substitutions that were fixed by natural selection (denoted α) can be estimated using extensions of the McDonald-Kreitman test [16], which compares non-synonymous and synonymous changes, and contrasts within-species polymorphism to fixed differences between species. We have extended existing maximum likelihood approaches [15],[23],[24] to estimate separate α values for immunity and non-immunity genes, and for different classes of immunity genes (see Materials and Methods).

We found that the proportion of substitutions attributable to positive selection in immune genes is approximately 50% greater than the genome average. Based on the divergence between *D. simulans* and *D. melanogaster* and polymorphism in Kenyan populations of both species, we estimated that 65% of amino acid substitutions in immunity genes have been fixed by selection (95% bounds bootstrapping across genes within categories: 55–72%, Figure 1A). This is significantly higher than our estimate for non-immunity genes, which is very close to previous genome-wide estimates (reviewed in [10]) (α = 41%; 95% bounds are 31–50%; difference from immunity genes: *p* = 0.004, inferred by bootstrapping).

The effect remained highly significant when data from all populations were combined, though absolute estimates of α were slightly lower (immune: α = 58%; non-immune: α = 33%; *p* = 0.004; Figure S10). Since the exclusion of rare variants led to slightly higher estimates of α (Figure S16), this effect is probably caused by the enlarged sample size containing a higher proportion of (low-frequency) mildly-deleterious non-synonymous variants, which can cause α to be underestimated [23]. Estimates of α in the Greek (Athens) populations had greater variance and failed to detect a significant difference between immunity and non-immunity genes (Figure S10B), as might be expected because the relatively low genetic diversity of this population means we have little statistical power to accurately infer α [14].

The proportion of amino acid substitutions fixed by selection (α) will clearly be affected by the number of substitutions not fixed by selection, i.e., the number of effectively neutral substitutions fixed through genetic drift. Therefore, it is possible that the higher α of immunity genes does not reflect any increase in the absolute number of adaptive substitutions per non-synonymous site (denoted *a* [16]). This possibility has been little explored, because *a*, unlike α, is difficult to estimate as a multi-gene average, and because single-gene estimates of either statistic tend to be imprecise. Here we use an approach that allows us to obtain relatively stable estimates of *a* for individual genes (see Materials and Methods), which can then be averaged across immune and non-immune genes. Using Kenyan populations of *D. melanogaster* and *D. simulans*, we estimated that since their common ancestor, selection has fixed an average of 10.6×10^−3^ adaptive substitutions per non-synonymous site in immunity genes, but only 5.7×10^−3^ in other genes (difference between immunity and control genes: *p* = 0.02; Figure 1B). This difference in the absolute number of adaptive substitutions corresponds to 50% increase in the proportion (α) described above, and suggests that natural selection is fixing adaptive substitutions in immunity genes at nearly double the genome average rate.

### Immune genes show more variation in rates of adaptive evolution than other genes

The high rate of adaptive evolution that we found in immunity genes could be driven either by a general elevation in the strength of selection across all immunity genes, or by a few key genes experiencing intense selection pressures. To investigate this, we examined the distribution of *a* across genes. Although mean *a* is higher for immunity genes than other genes (Figure 1B), the modal class is the same, i.e., centred on zero in both cases (Figure 2A *versus* Figure 2B), and the difference in mean is driven by a subset of immune genes with unusually high *a* (Figure 2C; this results in a significantly higher variance for immunity genes). The wider distribution of *a* across immunity genes suggests that most of these genes experience similar selection pressures to the rest of the genome, while a small subset are under substantially stronger selection. This is consistent with the analyses of *D. simulans* genome sequences that found little evidence that immunity genes as a group are outliers in terms of recurrent adaptive evolution [17]. Thus it appears that host-parasite arms races may involve a relatively small subset of the immune system.

This analysis could be confounded if our estimates were less accurate for immune genes than control genes, but this is unlikely for two reasons. First, the immunity genes tend to be longer than control genes, which will reduce the variance of *a* estimates and make our analysis conservative (Figure 2C). Second, the pattern remains significant and quantitatively almost identical if the analysis is restricted to genes with more than 500 non-synonymous sites (Figure S17, S18).

### Immune genes with different functions show different rates of adaptive evolution

Clues as to the nature of the selection pressures acting on immune genes can be gained from looking at which functional classes of immune gene are experiencing the strongest selection [1],[2]. To examine how selection pressures differ between immune genes with different functions, we classified the genes in two different ways.

First, we classified genes according to the branch of the immune system in which they function: the humoral, cellular, melanisation and antiviral RNAi responses. We found little variation between the first three categories (α = 51%, 62% and 63%; per-site *a* = 0.009, 0.010 and 0.012, respectively), and individually no category was significantly different from non-immunity genes (Figure 1A and Figure 1B). However, RNAi genes were an exception to this, showing approximately twice the proportion of adaptive substitutions as compared to non-immune genes (α = 88% *vs.* 41%; *p*<0.001), and seven times the number of adaptive substitutions per site (*a* = 0.042 *vs.* 0.0057; *p*<0.001; Figure 1). This is consistent with previous results, which found that some RNAi genes evolve rapidly under positive selection [6],[25].

Second, we classified immune genes (excluding those involved in RNAi) according to their mode of action: pathogen recognition, signalling cascade, and antimicrobial peptides (AMPs). This categorisation gave a superior fit to the data according to model selection techniques (see Materials and Methods, and Table S2) and was also a significantly better fit than randomly assigning genes to categories of the same size (randomization test: *p*<10^−3^). Using this alternative categorisation, no group was significantly higher than non-immune genes, although signalling molecules did have a marginally higher α but not *a* (estimated α = 57% *vs.* 41%; *p* = 0.085). Consistent with previous results [26],[27], AMPs showed no evidence of adaptive evolution (were not detectably different from α = 0; Figure 1A), undergo significantly less adaptive evolution than RNAi, signalling and cellular recognition genes (*p*<0.014 in each case), and undergo marginally less adaptive evolution than non-immune genes (estimated α = −13% *vs.* 41%; *p* = 0.082). Alternative analyses using other populations and outgroups resulted in a qualitatively identical pattern (Figures S10, S11, S12, S13, S14, S15), except that the use of *D. yakuba* as an outgroup resulted in the signalling molecules having a significantly higher α than the controls (*p*<0.031; Figure S14A and S14B).

### Some genes and pathways are under exceptionally strong selection

Because the high rate of adaptive evolution in immune system genes is caused mainly by a subset of genes under very strong selection (Figure 1 and Figure 2), we investigated how these genes are distributed across the immune system (Figure 3). The two main signalling pathways in the immune system are the Toll and IMD pathways, and of these the IMD pathway has a much higher rate of adaptive evolution than the Toll pathway (IMD: mean estimated *a* = 0.023; Toll: mean *a* = 0.009; difference between Toll and IMD *p* = 0.039 by bootstrapping within classes). Within the Toll pathway, the extracellular molecules are under stronger selection than the cytoplasmic ones (extracellular: mean *a* = 0.015, cytoplasmic: mean *a* = 0.005, *p* = 0.033). The antiviral RNAi genes again show strong adaptive evolution [6] (mean estimated *a* = 0.032). Elsewhere, TEP I and PGRP-LD are also under exceptionally strong selection [1],[7]. It has been suggested that the phagocytosis receptor *Dscam*, which can produce up to 18,000 differently spliced isoforms, may allow *Drosophila* to mount specific immune responses [28],[29]. However, despite having over 22 kbp of coding sequence from *Dscam*, we were unable to find any evidence of adaptive evolution in this gene, indicating that this gene is not subject to arms-race selection.

### Genes experience correlated selection pressures in different species

If the immune system adapts to parasites in similar ways in related species, then we would expect to see the same genes experiencing positive selection in different lineages [30]. Alternatively, each species could respond differently, resulting in different genes being positively selected in different lineages [30].

To address this question, we estimated the rate of adaptive evolution separately for each of the lineages leading to *D. simulans* and *D. melanogaster* from the common ancestor of the two species. The pattern of α (and *a*) across different pathways and functional categories of genes was very similar between the two lineages (Figures S12, S13), suggesting that the broad distribution of selection pressures between immune functions is the same. For example, in both lineages antiviral RNAi genes have the highest rates of adaptive evolution and antimicrobial peptides have the lowest rates.

Estimates of *a* along these individual lineages are associated with high levels of noise due to the short length of the branches; furthermore, the measurement error will be negatively correlated across the two lineages. Despite these sources of error, however, the data show a significant positive correlation in immunity gene *a* estimates between the two lineages (Figure 4), and this suggests that individual genes, and not just categories of gene, are under similar selection pressures in both lineages. This correlation was not significantly different to that that found in the non-immunity genes, indicating that there is no greater tendency for parasites to cause lineage specific selection than other selective agents (Figure 4).

### Immunity genes have similar levels of polymorphism and population structure to other genes

The analyses presented above can identify selection that has occurred over millions of years, but recent selective sweeps can also be detected though reductions in genetic diversity. In both *D. melanogaster* and *D. simulans* there was no significant difference in the diversity of synonymous sites (π_s_) between immunity and non-immunity genes (Kenyan *D. melanogaster*: π_s_ = 1.60% *vs.* 1.55%; Kenyan *D. simulans*: 2.46% *vs.* 2.62%; Figure S19, Figure S20, Table S3). Furthermore, if the immune genes are split into functional categories, only the diversity of the antiviral RNAi genes is significantly lower than the control genes (*D. melanogaster* π_s_ = 0.80%, *p*<0.001; *D. simulans* π_s_ = 1.01%, *p*<0.001. Figure S19, Figure S20, Table S3). This is consistent with RNAi genes having the highest rates of adaptive substitution in the immune system, and suggests a high proportion of them may have recently experienced selective sweeps in both species. Furthermore, none of the immune genes had unusually high levels of polymorphism, suggesting host-parasite coevolution in *Drosophila* has not resulted in the ancient polymorphisms like those seen in vertebrate MHC genes and some plant resistance genes [31],[32].

It is known that flies are infected by different parasites in different populations, and this could lead to local adaptation where different alleles of a gene are favoured in different populations [33],[34],[35],[36],[37]. However, we could not detect any differences between immune genes and the controls in the amount of population structure in either *D. melanogaster* or *D. simulans* (Figure S21) providing no evidence to suggest that local adaptation of immune genes is common. However, it should be noted that our statistical power to detect genetic structure may be extremely low, and the effects of local adaptation on patterns of nucleotide variation may be small [38].

We also compared the amino acid diversity (π_a_) of the immunity and control genes, as this may reflect differences in selective constraint or the effects of balancing selection. In all eight populations π_a_ was slightly higher in the immune genes, and in three populations the difference was significant (Figure S22, Figure S23, Table S3). Compared to the control genes, immune signalling molecules tend to have lower amino acid diversity, while antimicrobial peptides and recognition molecules in the cellular immune system have significantly higher amino acid diversity (Figure S22, S23). These differences correspond to the estimated number of substitutions occurring by genetic drift (Figure S24), but not to differences in π_s_, implying that they are caused by differences in selective constraint, rather than long-term balancing selection maintaining amino acid polymorphisms.

## Discussion

We have found that the rate of adaptive substitution in immunity genes is nearly double the genome average. This is the first quantitative estimate of the rate at which natural selection drives protein evolution in genes of the immune system relative to the genome as a whole, and confirms that adaptation to parasites is an important force driving evolution. There are several reasons why parasites may be a powerful selection pressure. Firstly, parasites can cause high rates of mortality and morbidity, and therefore have a large impact on the fitness of their hosts. Secondly, the direction of parasite-mediated selection continually changes, due to coevolutionary arms races between hosts and parasites [39], and ecological factors altering the composition of the parasite community. Finally, parasites generally have shorter generation times, and (in the case of viruses) elevated mutation rates, potentially giving them an edge in the ‘arms-race’. This means that hosts may often be maladapted to their current set of parasites, and therefore under strong selection to evolve resistance.

We have also found that the high rate of adaptive substitution of immunity genes is driven by a small subset of immune genes under strong selection, while the majority of immunity genes have similar rates of adaptive evolution to the rest of the genome. This suggests that rapid ‘arms-race’ coevolution may only involve a small subset of molecules in the immune system. Since there is a tendency for these strongly-selected genes to cluster by pathway or protein-family, these clusters may reflect hotspots for coevolutionary interaction with parasites.

By examining the function of these groups of strongly-selected genes, we can gain clues regarding the underlying molecular processes that drive this coevolution. It is striking that almost all of these genes fall within the IMD signalling pathway and the antiviral RNAi pathway (Figure 3). It is known that both signalling pathways and RNAi are targeted by parasite molecules that suppress the immune response, and it has been suggested that this suppression may cause much of the adaptive evolution seen in immunity molecules [1],[2],[4],[25],[40]. The Toll pathway tends to have lower rates of adaptive evolution. It is unclear why this is, although it may reflect the pathogens with which it interacts, or constraint from its other functions in development [41]. In contrast to the signalling pathways, the PGRPs and GNBPs that act as receptors for the Toll and IMD pathways are not positively selected, possibly reflecting their role in binding to highly conserved pathogen molecules [7]. Unlike many other organisms (especially vertebrates [42]), AMPs in *Drosophila* show less adaptive evolution than most genes. This contrasts with the high rate of AMP gain and loss in the *Drosophila* phylogeny [1], and suggests that whatever process favours the duplication of AMPs does not result in strong selection on their protein sequence. Our results also imply that AMPs may be weakly constrained, with genetic drift fixing amino acid substitutions at a relatively high rate. This may be a consequence of gene duplication, as duplicated genes often have elevated rates of amino acid substitution [43].

It is interesting to note that components of the antiviral RNAi pathway also mediate defence against transposable elements [44],[45],[46], and these ‘genomic parasites’ may be an important selective force on these genes [25]. Indeed, several RNAi genes with no reported anti-viral function [25],[47],[48], and other genes involved in chromatin function [17], show evidence of rapid adaptive evolution in *Drosophila*.

At the phenotypic level, many organisms show evidence of convergent evolution, with different species evolving similar adaptations in response to similar selection pressures. However, it is unclear whether convergence is also common in molecular evolution, or whether molecular evolution is idiosyncratic, with each species following a unique evolutionary pathway [30]. One way to address this question is to test whether the same genes are evolving adaptively in different species [30]. At a broad level, we found that similar functional classes of immunity genes tend to have elevated rates of adaptive evolution in both the *D. melanogaster* lineage and the *D. simulans* lineage. At a finer scale, the rate of adaptive evolution of individual genes is correlated in the two lineages (despite the very high levels of noise associated with these single-lineage estimates). Because this correlation was not significantly different in immunity genes and our control genes, this suggests the fluctuating selection pressures associated with host-parasite coevolution do not result in unusually high rates of lineage-specific selection. Together these results suggest that the immune system of these two closely related species experience similar selection pressures, and adapt to those selection pressures in similar ways.

Previous studies on immunity genes have applied various tests of adaptive evolution, and found that a higher than average fraction of immunity genes test ‘positive’ (e.g., [1],[2]). However, the statistical power of these tests will depend on factors such as selective constraint and gene length, and these could differ between immunity and non-immunity genes, even if their rates of adaptive substitution were identical. Furthermore, such confounding factors will be even more important if adaptive substitution is frequent across the genome, meaning that a large proportion of all genes evolve under some degree of positive selection [10]. Therefore a particular strength of the current approach, which can compare the estimated rates of adaptive evolution across different groups of genes, is that it provides quantitative estimates of the effect size rather than simply counting the number of ‘significant’ tests.

Estimates of the rate of adaptive substitution based on the McDonald-Kreitman test have been subject to some recent criticism as they can be influenced by factors such as population demography [18],[19]. However, it seems unlikely the differences observed here are artefacts. First, we compared loci where we have a strong *a priori* expectation of adaptive substitution to position-matched control loci. Second, we found no significant differences in the rate at which genetic drift causes non-adaptive evolution at these loci, such as could mislead the tests (Figure S24). Finally, false signatures of adaptive substitution can occur in populations that have experienced bottlenecks or recent expansions, and yet the signal we observed was much stronger in the ancestral Kenyan populations (Figure S10A), and weakest in the more derived populations (Figure S10B), while quantitative estimates of *a* differed surprisingly little between datasets. As new sequencing technologies result in ever larger datasets, this approach promises to be a powerful way to identify the selection pressures driving molecular evolution.

Our data not only confirm that parasites are an important driving force in molecular evolution [1],[2], they quantify the magnitude of this effect, and show that the rate of adaptive protein evolution in immunity genes is nearly twice the genome average. This elevated rate in the immune system is due to a subset of genes evolving under intense positive selection, and many of these genes are strongly selected in both *D. melanogaster* and *D. simulans*, suggesting that our results may reveal general principles of immune system evolution. In particular, some of the most strongly selected genes may be targeted by parasite suppressors the immune response, and this may be a key battlefield in coevolution. These data add to the growing evidence that much adaptive protein sequence evolution is driven by co-evolutionary conflicts within or between genomes [49],[50].

## Materials and Methods

### Sequencing and sequence analysis

Flies were sampled from six populations of *D. melanogaster* and two populations of *D. simulans*, covering both their original range in Africa and more recent global expansion. In each population we extracted genomic DNA from four female flies that were either collected from the wild or were the progeny of crosses between pairs of isofemale lines (i.e. we sampled eight chromosomes from each population). Targeted genes were amplified by PCR in ∼5 kbp products, and the PCR products from each population were then mixed together, purified on a gel, and sequenced using the Solexa-Illumina sequencing platform to high coverage (mean >130-fold; Figure S1). The 36 bp sequencing reads were aligned to the *D. melanogaster* or *D. simulans* genome using MAQ [51] allowing for up to 2 mismatches per read, which resulted in 5–16 million mapped reads in each population. The sites were then assigned to coding or non-coding sequence using the genome annotation, and coding sites were classified as synonymous or non-synonymous. Positions with less than 20-fold coverage were excluded, as were genes represented by less than 100 bp; however, our results were not strongly affected by the exclusion of sites with less than 50-fold or 100-fold coverage (Figure S25). Full details of the Solexa-Illumina sequencing, together with a detailed comparison with traditional Sanger sequencing, are given in Text S1. A full listing of loci, their positions and polymorphism counts are given in Table S1.

### Adaptive substitutions

To estimate the rate of adaptive substitution, we used a multi-locus, maximum likelihood extension of the McDonald-Kreitman test. This method is based on Welch 2006 (ref. [15], see also [23],[24]), but contains several new features and models. Software that implements the new methods is available on request from the authors, or from http://tree.bio.ed.ac.uk/software/.

We compared non-synonymous and synonymous divergence between *D. melanogaster* and *D. simulans* with polymorphism from both species. For each locus, the six observations (*d_N_*, *d_S_*, and *p_N_* and *p_S_* for each species), were assumed to have the following expected values:
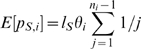


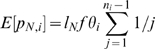


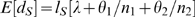


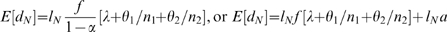
where *l_S_* and *l_N_* are the number of synonymous and non-synonymous sites, λ = μ*t* is the expected neutral divergence between the species, θ*_i_* = 4*N_e_*μ is the expected neutral polymorphism for species *i*, *n_i_* is the number of alleles sampled for species *i* (taken here to be 8 per sampled population), and *f* is the fraction of non-synonymous mutants that are effectively neutral [15].

The parameters of greatest interest here, α or *a*, quantify the multiplicative or additive deviation of the observed *d_N_* from its expectation under neutrality and purifying selection. Positive estimates of either α or *a* are consistent with adaptive protein evolution, while negative values result either from sampling error, or from the presence of mildly deleterious mutations (which violate the assumptions of the test, contributing to *p_N_* but rarely reaching fixation [16],[52]). This violation can be mitigated by excluding low frequency synonymous and non-synonymous polymorphisms, as this is expected to remove the great majority of mildly deleterious mutations while leaving the neutral *p_N_*/*p_S_* ratio unaltered [52],[53]. To explore this phenomenon, we repeated our analyses excluding all putative polymorphisms with an estimated minor-allele frequency below a range of threshold frequencies (Figure S3). Our results were qualitatively unaltered, and so in the main text we report only results with all sampled polymorphisms included in the counts.

To estimate the model parameters it was assumed that observed quantities were Poisson distributed around their expected values [15],[23],[24]. This distribution is derived under the assumption that substitutions and polymorphisms occur as independent events, but this assumption can be violated, e.g., by linked selection causing the clustering of substitution events in time. We used three approaches to reduce the impact of such violations. First, for some parameter types (selective constraint *f* and/or adaptive substitution *a*), we assigned separate parameters to each locus, making the extent of stochastic variation irrelevant to the parameter estimates obtained. Second, we obtained confidence intervals by bootstrapping across loci, rather than using the curvature of the likelihood surface. Third, we used model-selection criteria that allow for un-modeled over-dispersion (such as that arising from the clustering of events in time). To avoid over-parameterization associated with assigning large numbers of locus-specific parameters, we assumed that λ (the neutral mutation rate multiplied by divergence time) took a single value across all loci.

To model neutral polymorphism, we exploited the correlation between θ at a locus, and its local recombination rate [54], by fitting the model θ = *mr*+*b*, where *r* is the local *D. melanogaster* recombination rate [55]. Maximum likelihood estimates of *m* and *b* were then obtained for each of the two species. This model has the advantage of providing appropriate estimates of θ for loci where the synonymous polymorphism is not at equilibrium, such as after a recent selective sweep. Model selection techniques (see below) also showed that it was significantly preferred to models in which θ did not vary between loci, and in which each locus had a separate parameter. Importantly, however, estimates of *a* were very similar under all three parameterizations (Figure S26). Given our chosen model, a data set of *k* loci was used to fit *k*+5 nuisance parameters, plus the *a* or α values of interest.

To choose between different parameterizations of the likelihood model (see Table S2) we used the Akaike Information Criterion, corrected for finite sample size and over-dispersion in the count data [56]. This criterion is given by QAICc = −2lnL/*c*+2*K*+*K*(*K*+1)/(*n*-*K*-1) where lnL is the maximized likelihood for the model, *K* is the number of parameters it contains, and *n* is the number of data points (taken to be 6 times the number of loci). The factor *c* is the correction for overdispersion, and was estimated by *c* = (2lnL_full_-2lnL_sat_)/*n*_full_, where “full” denotes the largest model in the set of models being compared, and “sat” denotes the saturated model, in which the expected values of all data points were set to their observed values. The conditional likelihood of each model was obtained by converting the QAICc values into Akaike weights [56].

To compare estimates of adaptive substitution along two independent lineages, we used a variant of the method above, including polymorphism from a single species, and polarizing substitutions on to the *D. melanogaster* or *D. simulans* branch based on the inferred ancestral sequence. Ancestral sequences were inferred using maximum likelihood under a codon-based model and the tree (((Dmel,Dmel), (Dsim,Dsim)), ((Dyak), (Dere))) using PAML [57].

### Genetic diversity and differentiation statistics

Genetic diversity was quantified in two ways. First, an estimate of θ derived from the number of polymorphic sites, calculated exactly as Watterson's θ*_w_* under the assumption that all eight chromosomes in each population were sampled [58]. Although sites with low read depth may not sample all chromosomes, even at 20-fold coverage (our minimum threshold for inclusion) given equal representation of the chromosomes there is >90% chance that at least 7 of the 8 chromosomes have been sampled. Given the observed read depths this effect would lead us to underestimate Watterson's θ by less than 0.5% of its correct value for most loci (Figure S9). Second, an estimate of θ based on π (the average number of pairwise differences per site) was calculated from read frequencies (rather than allelic frequencies) at each site based on the assumption that read frequencies should reflect underlying allele frequencies. In fact, although significantly correlated, read frequencies do not provided a good estimate of allele frequencies in our data (Pearson's ρ = 71; Figure S4, see Text S1 for a full discussion). However, when averaged over multiple sites, *π* based on read-depth is extremely highly correlated with that based on true allele frequencies from Sanger sequence data, suggesting that this is an excellent measure of diversity (Pearson's ρ = 0.90; Figure S26).

The degree of population structure was quantified using a sequence-based estimate of *F_ST_* derived from π_s_ calculated within and between populations: *F_ST_* = (π_total_–π_sub_)/π_total_ [e.g. 59] where *π_sub_* is the average genetic diversity of a gene within a population and *π*_total_ is diversity across all populations. Averages across genes were calculated as the ratio between the mean of the numerator and the mean of the denominator for those genes, rather than the mean of the ratios. The significance of differences between classes of genes in *F_ST_* and genetic diversity was assessed by bootstrapping. Genes were re-sampled with replacement within each category, and the statistic was recalculated 1000 times to produce a null distribution.

## Supporting Information

Figure S1 The distribution of read depths. Histograms show the distribution of absolute read depths for every site analysed, separated into those inferred to be monomorphic (black) or polymorphic (blue; y-axis is relative frequency). Note that putatively polymorphic sites have a lower read depth, most likely because short-reads that differ from the reference are less likely to be successfully mapped to the genome. The x-axis shows the right hand limit of each bin, and read depths >499 are lumped at 500. The p-values report the probability that the two distributions are the same, based on a Kolmogorov-Smirnov test, as implemented in the R statistical language. The similarity between the two distributions suggests that the effect of polymorphisms on successful read-mapping is very small, and unlikely to qualitatively impact upon our conclusions.
(0.30 MB TIF)

Figure S2 Relative read depth as a function of position. Relative read depth by position (standardised to the population mean), plotted as the trimmed mean across 174 of the 5 kbp PCR products (this analysis excludes overlapping PCR products). To clearly illustrate end-effects, position is plotted from the centre of the fragment, and fragment lengths are standardised to 5 kbp by deleting sequences from the centre. Note that, on average, all samples show greater read depths at the ends of the fragment, and short regions of very low coverage ∼100 bp from the ends. This is likely to reflect poor fragmentation near PCR product ends and illustrates differences in fragmentation efficiency between samples.
(0.25 MB TIF)

Figure S3 Relative read depth as a function of polymorphic site density. Relative read depth for analysed sites (standardised to the mean for that population) is plotted against the number of inferred polymorphic sites in a surrounding 30 bp window, illustrating the reduced read depth in highly polymorphic regions. For clarity, only subsamples of the data are plotted, but the red lines show linear regressions calculated using all loci, with loci weighted equally. Read depths of <20-fold are set to zero. Because polymorphism and read depth are each positionally autocorrelated, simple regressions of read depth on the number of polymorphisms cannot be used to infer significance. Instead, *p*-values are derived from the distribution of per-gene point estimates of the correlation coefficient. In the absence of any underlying correlation, 50% of point-estimates for the ∼400 genes would be positive, and 50% negative. *P*-values report the probability of a deviation from 50∶50 that is as (or more) extreme than that observed, under a binomial distribution.
(0.23 MB TIF)

Figure S4 The relationship between Sanger and Solexa-Illumina estimates of allele-frequency. To assess the impact of short-read sequencing errors on estimates of minor allele frequency we re-sequenced 11 loci in the Greek (Athens) populations of *D. melanogaster* and *D. simulans* (see Text S1, Supplementary Methods). The relationship between minor allele frequencies estimated from Sanger sequences and from Solexa-Illumina sequences is shown for all polymorphic sites appearing in both datasets. Points are coloured according to read depth at that site; the solid line depicts a 1∶1 relationship, and the dashed line a linear regression of Solexa-Illumina on Sanger estimates. Note that because 8 chromosomes were sampled, the true minor allele frequency can only take values 1/8, 2/8, 3/8, 4/8. Although the correlation is relatively low (Pearson's rho = 0.71) this has surprisingly little impact of measures of diversity estimated using multiple sites (see Figure S9).
(0.18 MB TIF)

Figure S5 Relative read depth as a function of local GC content—all sites. Relative read depth for all sites are plotted against the GC content of a surrounding 200 bp window, illustrating how read depth is affected by local base composition. Data are derived from 174 ∼5 kbp long PCR fragments, excluding the 1,250 bp at each end to avoid end-based fragmentation effects (see Figure S9). Read depths are standardised to the sample mean, and subsamples of the data are plotted for clarity, but the red lines show linear regressions calculated across all 174 PCR amplicons weighted equally. Consistent with the analyses, read depths of <20-fold are set to zero. Because GC content and read depth are each positionally autocorrelated, *p*-values were calculated as in Figure S3. Note that the sign of the correlation changes between low read depth populations (Japan to Kenya) and higher read depth (French Polynesia and Florida) populations.
(0.40 MB TIF)

Figure S6 Relative read depth as a function of local GC content—analysed coding sites. Graphs are exactly as Figure S5, but include only the analysed (protein-coding) sequences (which have a higher average GC content). Relative read depth for analysed sites (standardised to the sample mean) is plotted against the GC content to illustrate how read depth is affected by local base composition. Because GC content and read depth are each positionally autocorrelated, simple regressions of read depth on local GC cannot be used to infer significance, therefore *p*-values were calculated as in Figure S3.
(0.36 MB TIF)

Figure S7 Base composition at variable sites. Bars show the base-composition of inferred polymorphisms (variants with a minor call-frequency of ≥5%; A, C, and E) and putative sequencing errors (variants with minor-call frequency <1%; B, D, and F) for all of the variable sites identified in the coding sequences. The y-axis is expressed as a proportion, and the x-axis denotes the major allele→minor allele change, i.e. A→C, G→C, etc. Note the large number of A→G and T→C amongst the inferred errors (B, D and F) relative to inferred polymorphisms (A, C, and E), which may be symptomatic of PCR-induced mutation. The effect is shown for Japan (A and B) which had lowest read-depth, Florida (C and D) which had highest read depth, and for all populations combined (E and F).
(0.39 MB TIF)

Figure S8 The relationship between Sanger and Solexa-Illumina estimates of diversity. To assess the impact of short-read sequencing errors on estimates of diversity we re-sequenced 11 loci in the Greek (Athens) populations of *D. melanogaster* and *D. simulans* (see Text S1 supplementary methods). (A) shows the relationship between θ_w_ estimated from Sanger sequences and from θ_w_ estimated from short-read sequences, (B) shows the same for average pairwise diversity (θ_π_). Triangles are loci re-sequenced in *D. simulans* and squares are loci re-sequenced in *D. melanogaster*; the solid lines depict a 1∶1 relationship, and dashed lines a linear regression of short-read estimates on Sanger estimates. Much of the difference between the two estimates is due to allelic dropout in *D. simulans* TepII, caused by a segregating indel at the site of the Solexa Long-PCR primer.
(0.17 MB TIF)

Figure S9 Underestimates of Watterson's θ_w_ due to un-sampled genomes. In calculating θ_w_ we assumed that all 8 chromosomes were sampled. However, at low coverage sites (<50-fold) it is unlikely that this is the case, and this could potentially lead to underestimates of θ_w_. We have calculated the effect of this on our estimates under the assumption that all the chromosomes are equally represented in the template pool and are sampled at random in the short reads. We find that the effect is small (A–E, below). Given our read depths for each locus in each population, we underestimate θ_w_ by <3% of the correct value at very low coverage and <0.5% at most loci. This is because (F) even at 20-fold coverage there is >90% chance of sampling 7 or 8 chromosomes, and the denominator of Wattersons's estimator (Σ^n-1^_i = 1_(1/i)) differs little between n = 7 and n = 8.
(0.35 MB TIF)

Figure S10 The estimated proportion of adaptive substitutions inferred by using polymorphism data from different populations. Graphs show the estimated proportion of amino acid substitutions fixed by selection (α) between *D. melanogaster* and *D. simulans* using data from different populations. (A) Kenyan populations only, based on 8 chromosomes of each (reproduced from the main text for comparison); (B) Greek populations only, based on 8 chromosomes of each; (C) All 8 populations (6 *D. melanogaster* and 2 *D. simulans*), based on 48 chromosomes of *D. melanogaster* and 16 chromosomes of *D. simulans*. Note that absolute estimates are smaller when all populations are used in the analysis, probably due to more rare variants. Error bars are 95% bootstrap intervals from re-sampling genes within classes; *p*-values are relative to the “control” genes, assessed by bootstrapping.
(0.27 MB TIF)

Figure S11 The correlation between estimates of a from different sample populations. The estimated number of adaptive substitutions per non-synonymous site (*a*) was little affected by the choice of population to provide polymorphism data. (A) shows the correlation in a between estimates using single Greek populations of *D. melanogaster* and *D. simulans*, and estimates using single Africa populations of *D. melanogaster* and *D. simulans* (both using *D. melanogaster*-*D. simulans* divergence) (Pearson's product-moment correlation  = 0.87, *p*<2×10^−16^). (B) shows the correlation between a single African population and all combined populations of both species (Pearson's product-moment correlation = 0.95, *p*<2×10^−16^). Thus estimates of a are similar when using African and non-African populations, and small African samples (8 chromosomes per species) provide almost the same information as global samples (48 *D. melanogaster* chromosomes and 16 *D. simulans* chromosomes). Immune genes are shown in red, and other genes in blue. Visually identified outliers are labelled.
(0.20 MB TIF)

Figure S12 The estimated proportion of adaptive substitutions inferred separately along the *D. melanogaster* and *D. simulans* lineages using Kenyan populations. By using *D. yakuba* and *D. erecta* to infer the state of the *D. melanogaster*-*D. simulans* common ancestor, substitutions can be assigned to the *D. melanogaster* or *D. simulans* lineage alone, and α inferred for each species separately. (A) *D. melanogaster* using a single Kenyan population only; (B) *D. simulans* using a single Kenyan population only. Note that immunity and control genes do not differ significantly, but this is probably due to the low power associated with the very small divergence. Interestingly, although the pattern across gene classes is qualitatively identical between species, absolute estimates of α are consistently higher in *D. simulans*. Error bars are 95% bootstrap intervals from re-sampling genes within classes, and p-values are relative to the control genes, assessed by bootstrapping.
(0.17 MB TIF)

Figure S13 The estimated proportion of adaptive substitutions inferred separately along the *D. melanogaster* and *D. simulans* lineages using all sampled populations. By using *D. yakuba* and *D. erecta* to infer the ancestral state of the *D. melanogaster*–*D. simulans* common ancestor, α was inferred separately for each species (see Figure S12 above). (A) *D. melanogaster* using polymorphism data from all six *D. melanogaster* populations; (B) *D. simulans* using both Kenyan and Athens populations. As in Figure S12, the immunity-control comparison is not significant for *D. melanogaster*, and estimates of α are consistently much higher in *D. simulans*. However, unlike Figure S12, here the effect of species is conflated with the number of sampled populations, and thus the presence of rarer alleles in *D. melanogaster*. Error bars are 95% bootstrap intervals from re-sampling genes within classes, and *p*-values are relative to the control genes, assessed by bootstrapping.
(0.17 MB TIF)

Figure S14 The estimated proportion of adaptive substitutions between *D. yakuba* and *D. melanogaster/simulans* using Kenyan populations. (A) *D. melanogaster* vs *D. yakuba*, using a single Kenyan population of *D. melanogaster*; (B) *D. simulans* vs. *D. yakuba* using a single Kenyan population *D. simulans*. Although the pattern across gene classes is qualitatively identical between species, absolute estimates of α are consistently higher in *D. simulans* (see also Figures S12, S13). Error bars are 95% bootstrap intervals from re-sampling genes within classes, and *p*-values are relative to the control genes, assessed by bootstrapping.
(0.17 MB TIF)

Figure S15 The estimated proportion of adaptive substitutions between *D. yakuba* and *D. melanogaster/simulans* using all sampled populations. (A) *D. melanogaster* vs *D. yakuba*, using all sampled *D. melanogaster* populations; (B) *D. simulans* vs. *D. yakuba* using both *D. simulans* populations. As in Figures S12, S13, S14, absolute estimates of α are consistently much higher in *D. simulans*, however, unlike Figure S14, here the effect of species is conflated with the number of sampled populations, and thus the presence of rarer alleles in *D. melanogaster*. Error bars are 95% bootstrap intervals from re-sampling genes within classes, and *p*-values are relative to the control genes, assessed by bootstrapping.
(0.20 MB TIF)

Figure S16 The estimated proportion of adaptive substitutions inferred by using polymorphism data from alleles that appear at different frequencies. Graphs show the estimated amount of adaptive substitution between *D. melanogaster* and *D. simulans*, based on polymorphism data from both species, for Kenyan populations only (A and B) and for all populations (C and D). (A and C) show the effect of excluding low-frequency alleles on α (the estimated proportion of adaptive substitutions) for classes of immune (red) and non-immune (blue) genes. Note there is a 5% frequency threshold per-population for inclusion in any of the analyses (See main text). (B and D) show the effect of excluding low-frequency alleles on a (the estimated number of adaptive substitutions per non-synonymous site) for immune (red) and non-immune (blue) genes individually. The solid grey line represents a 1∶1 correspondence, the dashed line a linear regression. The effect of excluding rare variants is both small, and consistent with theoretical expectations. This suggests that the presence of weakly-deleterious alleles that slightly depress estimates of α, but do not have a substantial impact upon our conclusions. It also suggests that our sequencing errors and inclusion-threshold have a minimal impact upon our conclusions.
(0.28 MB TIF)

Figure S17 The distribution of the number of adaptive substitutions (*a*) between genes, excluding short genes. Although mean *a* (the number of adaptive substitutions per non-synonymous site) is significantly higher for immune genes than for other genes, the modal class is similar and the variance larger (see main text). The greater variance in non-immunity genes could be attributed to shorter sampled gene length giving rise to greater sampling error. However, the exclusion of short genes from both classes does not alter the effect, as variance in immunity genes is still greater than that in non immunity genes (A–C); Var(*a*)×10^−4^ = 3.2 *vs*. 1.3, *p* = 0.0017. Immune genes are shown in red, and other genes in blue. Note that we used *a* in place of α for this per-gene analysis because α is poorly estimated for single genes (see Methods).
(0.12 MB TIF)

Figure S18 The distribution of the number of adaptive substitutions (*a*) between genes, using only genes intentionally targeted by PCR. The greater variance in non-immunity genes (see Figure S17) might also be attributed to shorter gene length giving rise to greater sampling error. In our primary dataset there are a large number of short gene fragments from non-immunity genes that appear in our sample merely because they happened to occur within the amplicons of a “targeted” gene. However, the exclusion of these “un-targeted” genes does not alter the effect. Variance in immunity genes is still greater than non immunity genes (A–C); Var(*a*)×10^−4^ = 4.8 *vs.* 2.3, *p* = 0.0177. Immune genes are shown in red, and other genes in blue.
(0.12 MB TIF)

Figure S19 Neutral diversity in *D. melanogaster*. Genetic diversity at synonymous sites in immunity and non-immunity genes (π_s_). Note that we do not have direct estimates of allele frequency (see Methods), and instead we use read frequency as a surrogate to calculate π. However, results based on Watterson's θ were very similar, and our estimates of π_s_ and θ_w_ were very highly correlated (r^2^>0.95 in each population). (A) Kenya; (B) Athens; (C) All *D. melanogaster* populations combined. Error bars are 95% bootstrap intervals of the mean from re-sampling genes within classes, and *p*-values are relative to the control genes, assessed by bootstrapping.
(0.22 MB TIF)

Figure S20 Neutral diversity in *D. simulans*. Genetic diversity at synonymous sites in immunity and non-immunity genes (π_s_). (see Figure S19 for details). Again, results based on Watterson's θ were very similar, as our estimates of π_s_ and θ_w_ were very highly correlated (r^2^>0.93 in each population). (A) Kenya; (B) Athens; (C) Both populations combined. Error bars are 95% bootstrap intervals of the mean from re-sampling genes within classes, and *p*-values are relative to the control genes, assessed by bootstrapping.
(0.24 MB TIF)

Figure S21 Genetic differentiation (*F_ST_*) between populations. Genetic differentiation between populations (*F_ST_*) at synonymous-sites in immunity and non-immunity genes. Error bars are 95% bootstrap intervals of the mean from re-sampling genes within classes, and *p*-values are relative to the control genes, assessed by bootstrapping.
(0.18 MB TIF)

Figure S22 Non-synonymous diversity in *D. melanogaster*. Genetic diversity at non-synonymous-sites in immunity and non-immunity genes (π_a_). (A) Kenya; (B) Athens; (C) All *D. melanogaster* populations combined. Error bars are 95% bootstrap intervals of the mean from re-sampling genes within classes, and *p*-values are relative to the control genes, assessed by bootstrapping.
(0.22 MB TIF)

Figure S23 Non-synonymous diversity in *D. simulans*. Genetic diversity at non-synonymous-sites in immunity and non-immunity genes (π_a_). (A) Kenya; (B) Athens; (C) Both populations combined. Error bars are 95% bootstrap intervals from re-sampling genes within classes, and *p*-values are relative to the control genes, assessed by bootstrapping.
(0.23 MB TIF)

Figure S24 The estimated number of non-adaptive substitutions per site between *D. melanogaster* and *D. simulans*. The estimated number of substitutions per non-synonymous site that were driven by genetic drift is shown. This number was estimated from (*D_n_/L_n_*)-*a*, where *a* is the estimated number of adaptively-driven substitutions; note that when a is separately parameterized at each locus, this removes from the estimates any dependency on the observed *D_n_* values. The estimates of drift-mediated substitutions are less variable within categories of locus than are estimates of adaptive substitution (although this must be partly due to the lack of dependence on the observed *D_n_* decreasing error variance). There are also fewer significant differences between classes of locus, notably a lack of difference between immunity and control genes. (A) Kenyan populations only; (B) All 8 populations (6 *D. melanogaster* and 2 *D. simulans*). Error bars are 95% bootstrap intervals from re-sampling genes within classes, and *p*-values are relative to the control genes, assessed by bootstrapping.
(0.18 MB TIF)

Figure S25 The estimated proportion of adaptive substitutions (α) between *D. melanogaster* (Kenya population) and *D. yakuba* according to read depth. Limiting the analysis to sites of high depth of coverage (>50-fold, >100-fold) has little impact on inferred rates of adaptive evolution.
(0.28 MB TIF)

Figure S26 Single-gene estimates of *a* using different models. To estimate single-gene *a*-values we fitted a model in which the parameter θ = 4Nμ was shared between loci as a linear function of recombination rate (see Methods). To explore the effect of this constraint, we compared our estimates of *a* to estimates derived using a single θ shared between all loci. (A) Pearson's correlation coefficient = 0.99, p<10–15), and separate estimates of θ for each locus. (B) Pearson's correlation coefficient = 0.75, *p*<10–15. In (B), the conspicuous outliers are almost all control genes that fell within the 5 Kbp amplicons, but which were not targets of primer design (see Text S1, detailed methods), and lack polymorphism data for *D. simulans*. This leads to over fitting at these loci when θ is a locus-specific parameter, and therefore poor estimation of *a*. In any case, use of the smaller model will tend to make our analyses conservative.
(0.22 MB TIF)

Table S1 Locations, classification, and genetic summary statistics for individual loci. Legend:- FBgn: FlyBase gene identifier. Locus: Locus name. Immune: Immune or Non-immune related. Class: Classified as Humoral, Cellular, RNAi, Melanisation, other immune, or Control. Cell_Hum: Classified as AMP, Humoral recognition, Cellular Recognition, Signalling, RNAi, other immune or Control. *a*: The estimated number of adaptive substitutions per site (method of Welch 2006). non-*a*: The estimated number of non-adaptive substitutions fixed by drift, per site. r: Local recombination rate in *D. melanogaster*. Ls: The number of synonymous sites. Ln: The number of non-synonymous sites. Dn: The number of non-synonymous fixed differences. Ds: The number of synonymous fixed differences. Mel_Pn: The number of non-synonymous polymorphisms in *D. melanogaster*. Sim_Pn: The number of non-synonymous polymorphisms in *D simulans* Mel_Ps: The number of synonymous polymorphisms in *D. melanogaster*. Sim_Ps: The number of synonymous polymorphisms in *D. simulans*. Position: Genomic position in the *D. melanogaster* genome release 5.7 Chromosome *p*-value: Fisher's Exact test *p*-value for a classical one-locus McDonald-Kreitman test FDR *q*-value: False-Discovery rate *q*-value, based on the distribution of *p*-values.
(0.26 MB XLS)

Table S2 Model selection. The table gives parameters relevant to model-selection between different parameterizations of between-locus variation in α (the estimated proportion of amino-acid substitutions driven by positive natural selection).
(2.70 MB PDF)

Table S3 Synonymous and non-synonymous diversity. Synonymous and non-synonymous diversity for different categories of gene in all populations, with those categories that were individually significantly different (*p*<0.05) from the control genes highlighted.
(2.41 MB PDF)

Text S1 Detailed supplementary methods.
(0.06 MB DOC)
