# Supplementary figures and images for: Quantifying Adaptive Evolution in the Drosophila Immune System
Source: PLoS Genet. 2009 Oct 23;5(10):e1000698. doi: 10.1371/journal.pgen.1000698 (PMC2759075; doi:10.1371/journal.pgen.1000698)

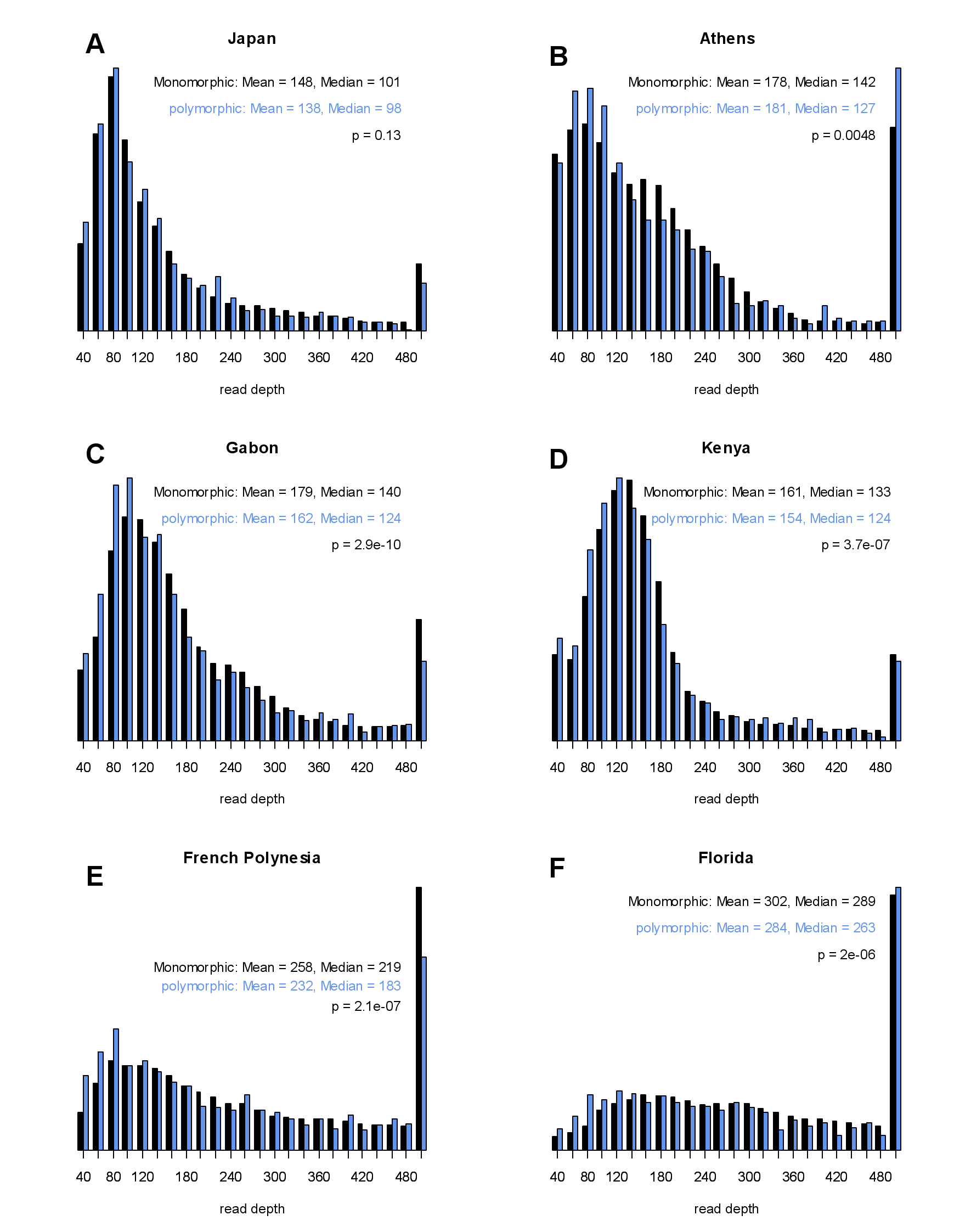

Supplement: Figure S1 — The distribution of read depths. Histograms show the distribution of absolute read depths for every site analysed, separated into those inferred to be monomorphic (black) or polymorphic (blue; y-axis is relative frequency). Note that putatively polymorphic sites have a lower read depth, most likely because short-reads that differ from the reference are less likely to be successfully mapped to the genome. The x-axis shows the right hand limit of each bin, and read depths >499 are lumped at 500. The p-values report the probability that the two distributions are the same, based on a Kolmogorov-Smirnov test, as implemented in the R statistical language. The similarity between the two distributions suggests that the effect of polymorphisms on successful read-mapping is very small, and unlikely to qualitatively impact upon our conclusions. (0.30 MB TIF) [file pgen.1000698.s001.tif]

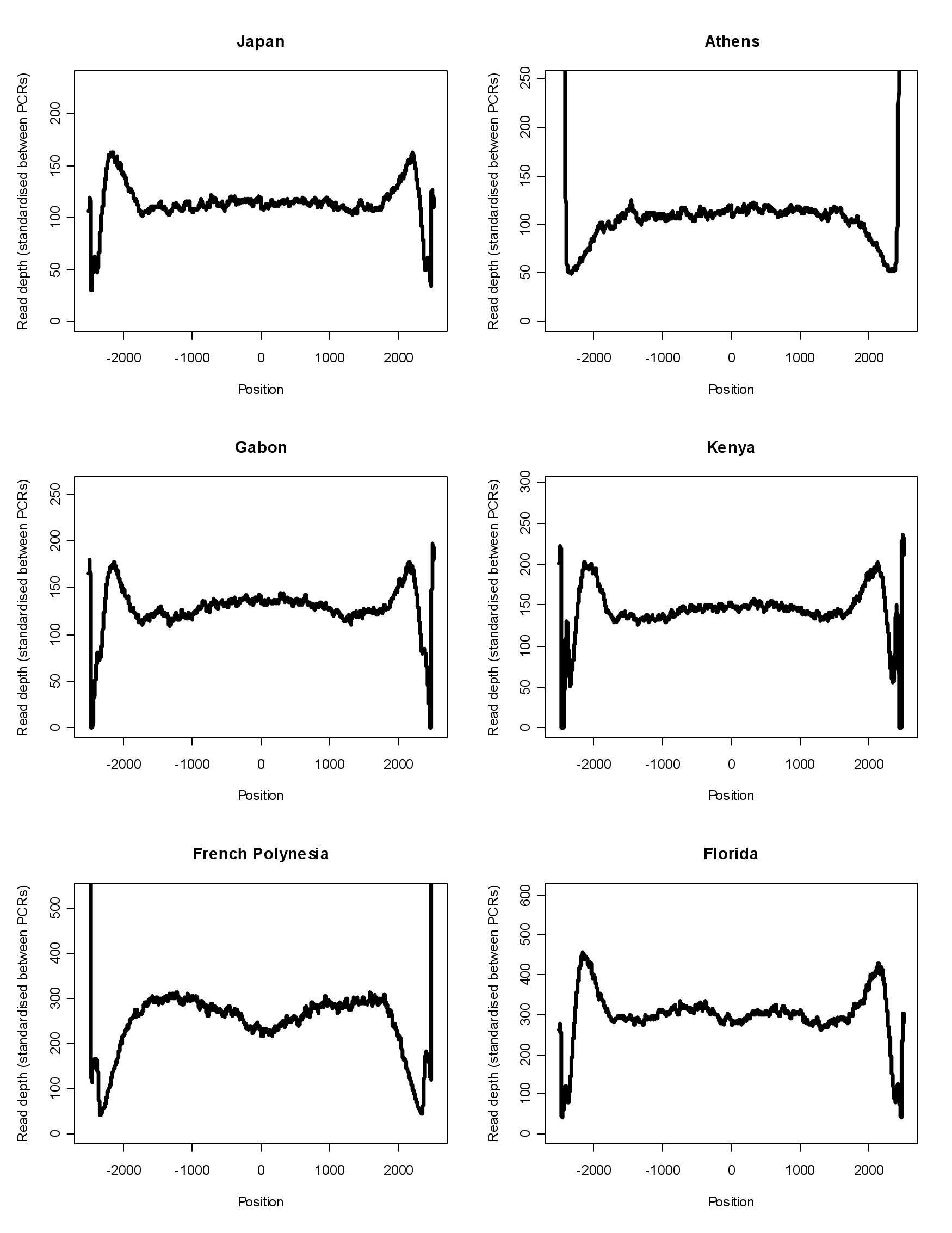

Supplement: Figure S2 — Relative read depth as a function of position. Relative read depth by position (standardised to the population mean), plotted as the trimmed mean across 174 of the 5 kbp PCR products (this analysis excludes overlapping PCR products). To clearly illustrate end-effects, position is plotted from the centre of the fragment, and fragment lengths are standardised to 5 kbp by deleting sequences from the centre. Note that, on average, all samples show greater read depths at the ends of the fragment, and short regions of very low coverage ∼100 bp from the ends. This is likely to reflect poor fragmentation near PCR product ends and illustrates differences in fragmentation efficiency between samples. (0.25 MB TIF) [file pgen.1000698.s002.tif]

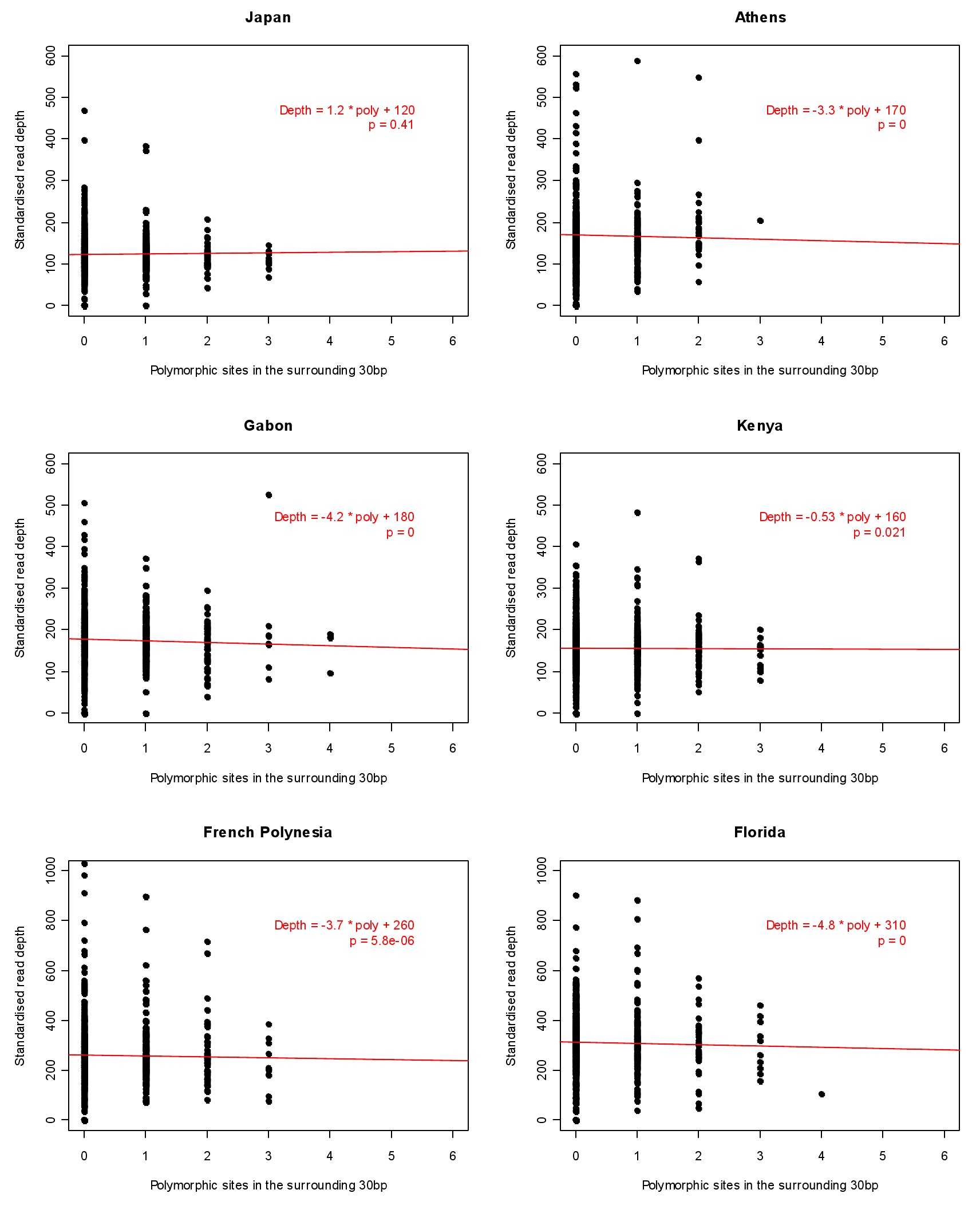

Supplement: Figure S3 — Relative read depth as a function of polymorphic site density. Relative read depth for analysed sites (standardised to the mean for that population) is plotted against the number of inferred polymorphic sites in a surrounding 30 bp window, illustrating the reduced read depth in highly polymorphic regions. For clarity, only subsamples of the data are plotted, but the red lines show linear regressions calculated using all loci, with loci weighted equally. Read depths of <20-fold are set to zero. Because polymorphism and read depth are each positionally autocorrelated, simple regressions of read depth on the number of polymorphisms cannot be used to infer significance. Instead, p-values are derived from the distribution of per-gene point estimates of the correlation coefficient. In the absence of any underlying correlation, 50% of point-estimates for the ∼400 genes would be positive, and 50% negative. P-values report the probability of a deviation from 50∶50 that is as (or more) extreme than that observed, under a binomial distribution. (0.23 MB TIF) [file pgen.1000698.s003.tif]

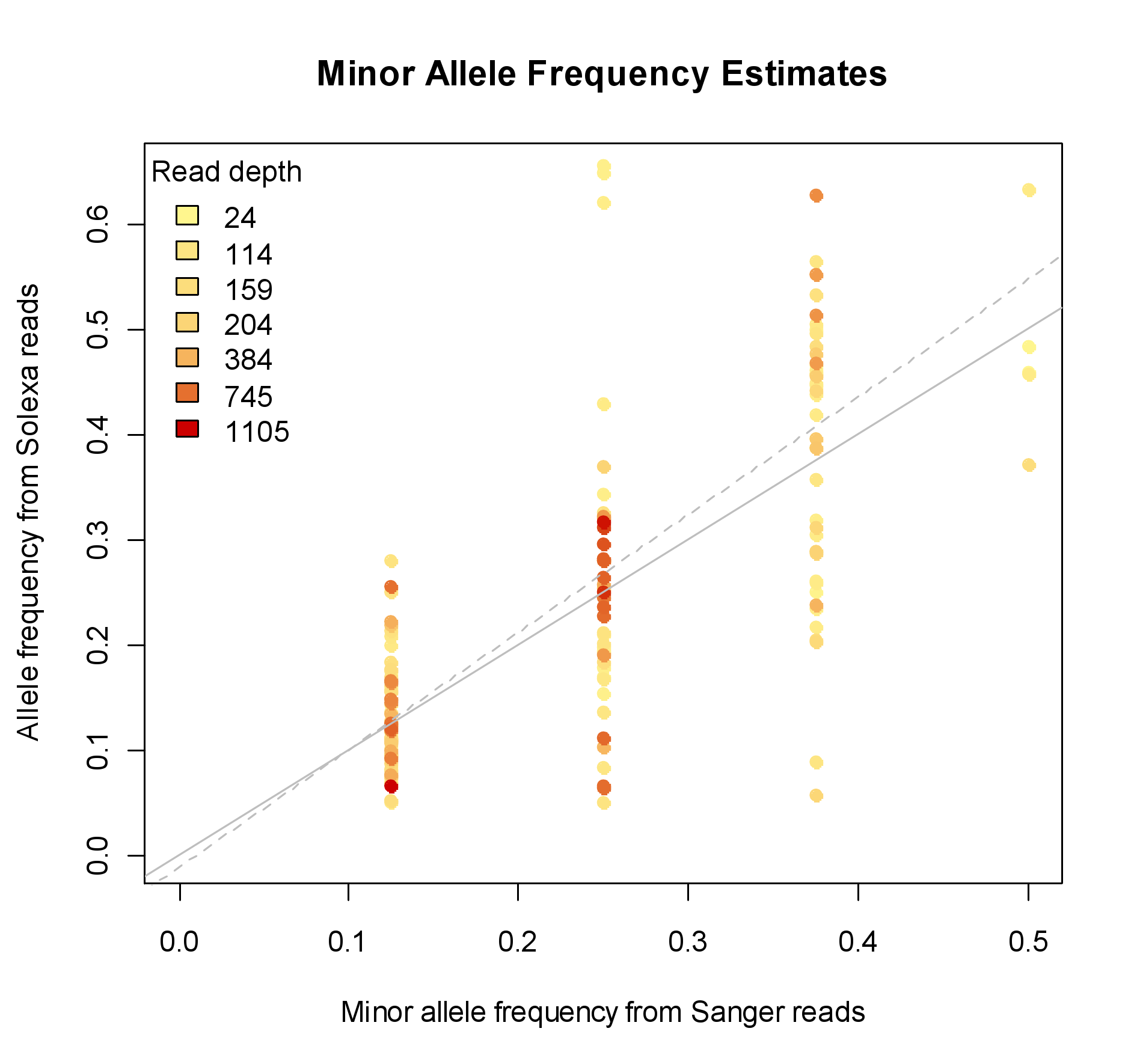

Supplement: Figure S4 — The relationship between Sanger and Solexa-Illumina estimates of allele-frequency. To assess the impact of short-read sequencing errors on estimates of minor allele frequency we re-sequenced 11 loci in the Greek (Athens) populations of D. melanogaster and D. simulans (see Text S1, Supplementary Methods). The relationship between minor allele frequencies estimated from Sanger sequences and from Solexa-Illumina sequences is shown for all polymorphic sites appearing in both datasets. Points are coloured according to read depth at that site; the solid line depicts a 1∶1 relationship, and the dashed line a linear regression of Solexa-Illumina on Sanger estimates. Note that because 8 chromosomes were sampled, the true minor allele frequency can only take values 1/8, 2/8, 3/8, 4/8. Although the correlation is relatively low (Pearson's rho = 0.71) this has surprisingly little impact of measures of diversity estimated using multiple sites (see Figure S9). (0.18 MB TIF) [file pgen.1000698.s004.tif]

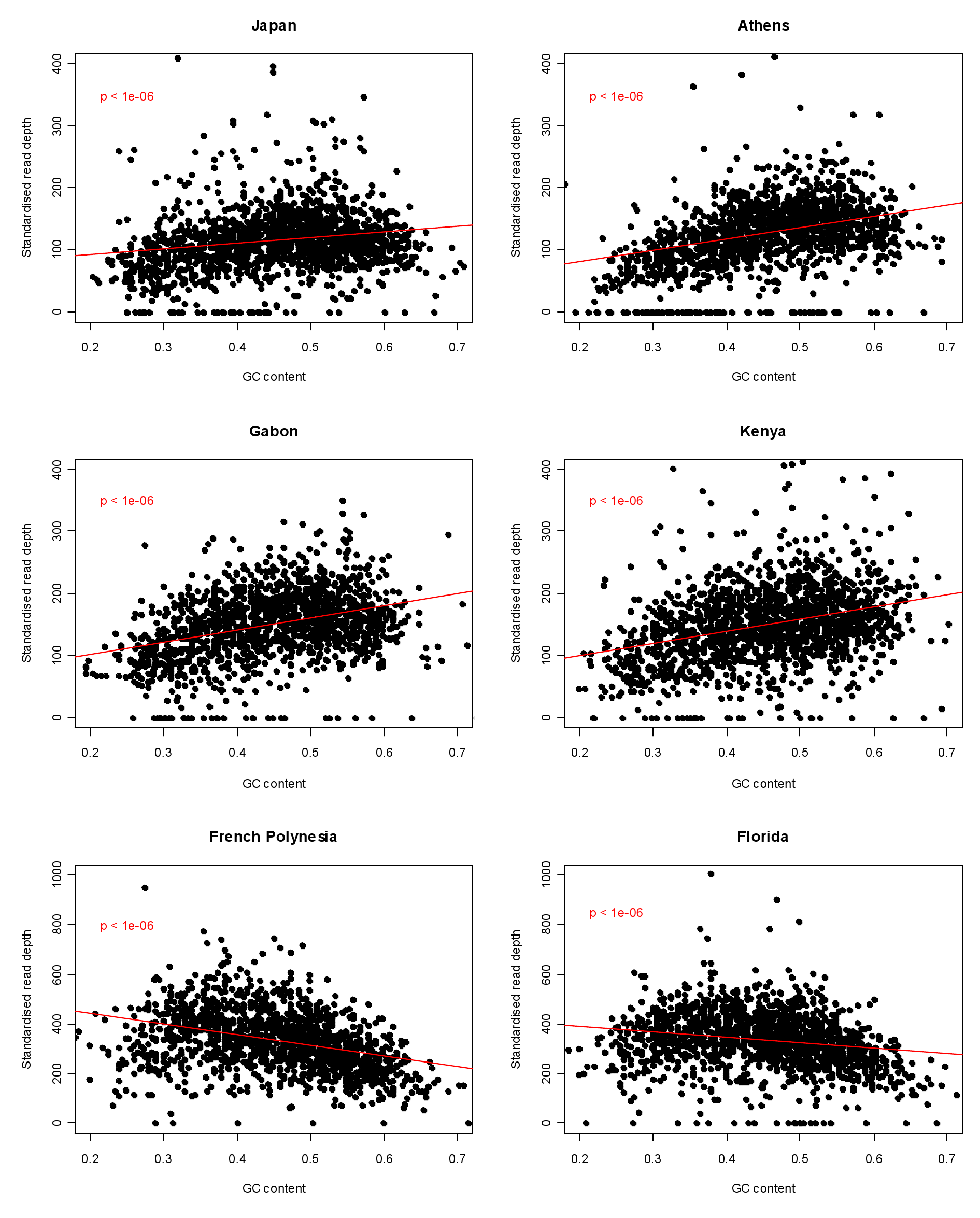

Supplement: Figure S5 — Relative read depth as a function of local GC content—all sites. Relative read depth for all sites are plotted against the GC content of a surrounding 200 bp window, illustrating how read depth is affected by local base composition. Data are derived from 174 ∼5 kbp long PCR fragments, excluding the 1,250 bp at each end to avoid end-based fragmentation effects (see Figure S9). Read depths are standardised to the sample mean, and subsamples of the data are plotted for clarity, but the red lines show linear regressions calculated across all 174 PCR amplicons weighted equally. Consistent with the analyses, read depths of <20-fold are set to zero. Because GC content and read depth are each positionally autocorrelated, p-values were calculated as in Figure S3. Note that the sign of the correlation changes between low read depth populations (Japan to Kenya) and higher read depth (French Polynesia and Florida) populations. (0.40 MB TIF) [file pgen.1000698.s005.tif]

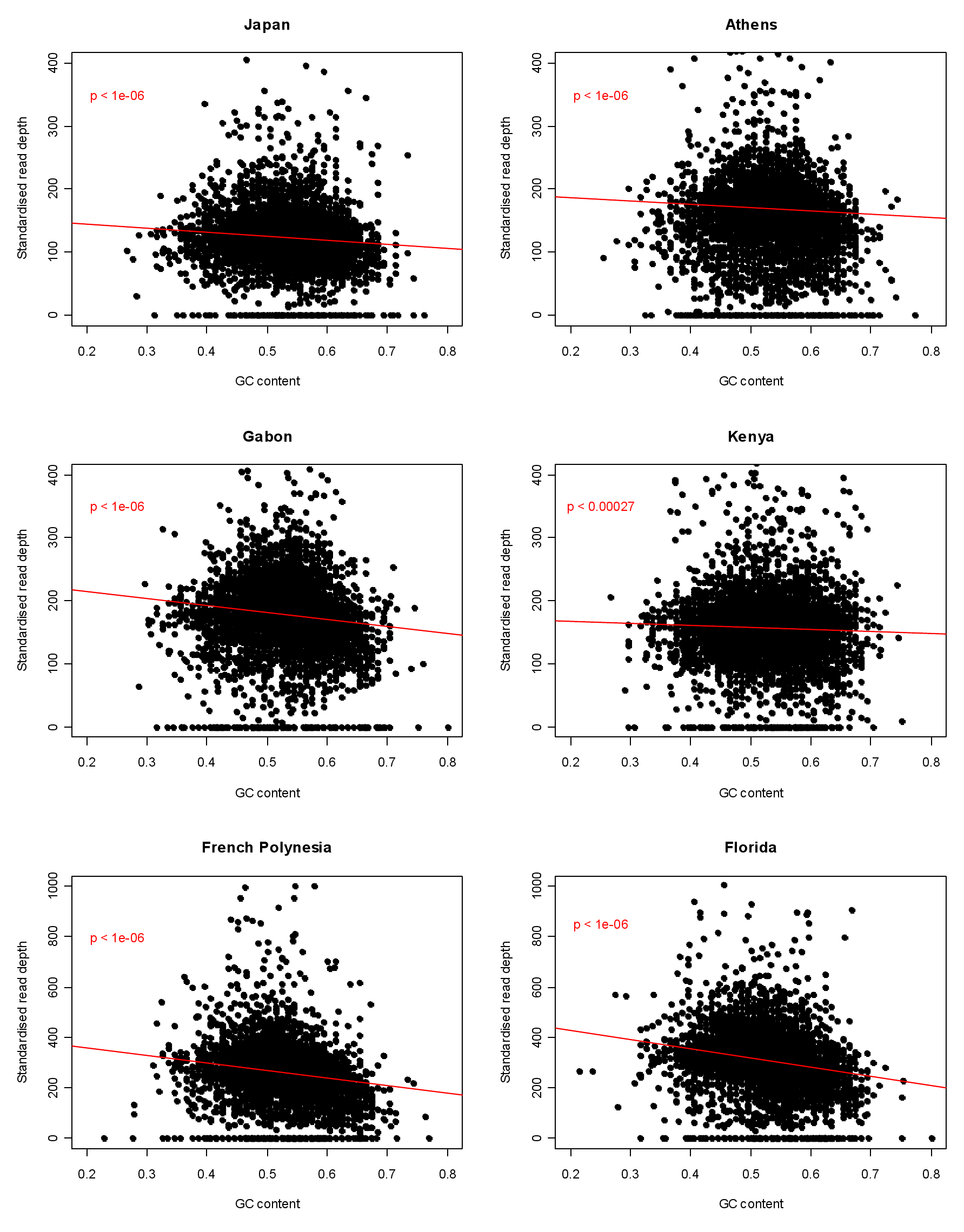

Supplement: Figure S6 — Relative read depth as a function of local GC content—analysed coding sites. Graphs are exactly as Figure S5, but include only the analysed (protein-coding) sequences (which have a higher average GC content). Relative read depth for analysed sites (standardised to the sample mean) is plotted against the GC content to illustrate how read depth is affected by local base composition. Because GC content and read depth are each positionally autocorrelated, simple regressions of read depth on local GC cannot be used to infer significance, therefore p-values were calculated as in Figure S3. (0.36 MB TIF) [file pgen.1000698.s006.tif]

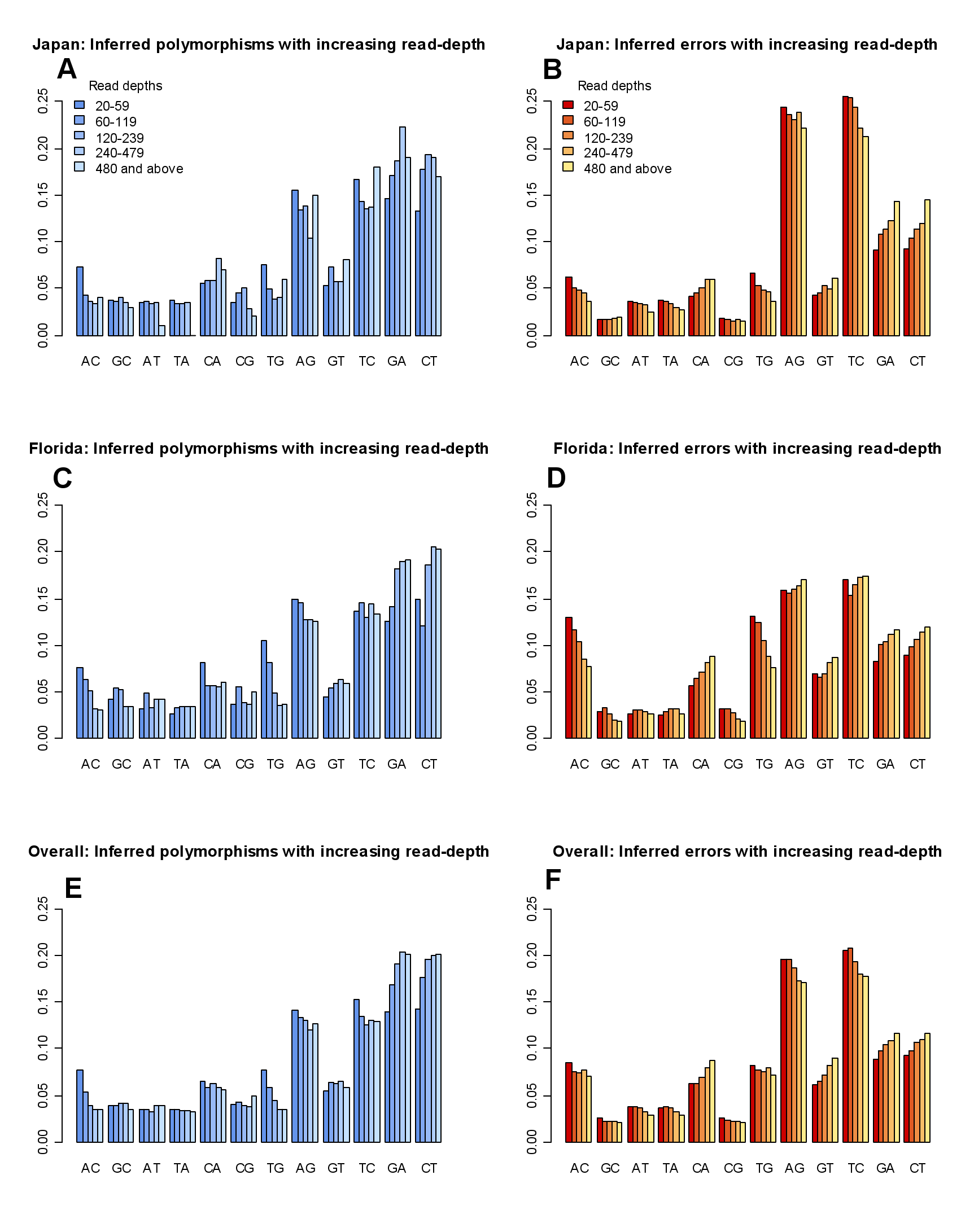

Supplement: Figure S7 — Base composition at variable sites. Bars show the base-composition of inferred polymorphisms (variants with a minor call-frequency of ≥5%; A, C, and E) and putative sequencing errors (variants with minor-call frequency <1%; B, D, and F) for all of the variable sites identified in the coding sequences. The y-axis is expressed as a proportion, and the x-axis denotes the major allele→minor allele change, i.e. A→C, G→C, etc. Note the large number of A→G and T→C amongst the inferred errors (B, D and F) relative to inferred polymorphisms (A, C, and E), which may be symptomatic of PCR-induced mutation. The effect is shown for Japan (A and B) which had lowest read-depth, Florida (C and D) which had highest read depth, and for all populations combined (E and F). (0.39 MB TIF) [file pgen.1000698.s007.tif]

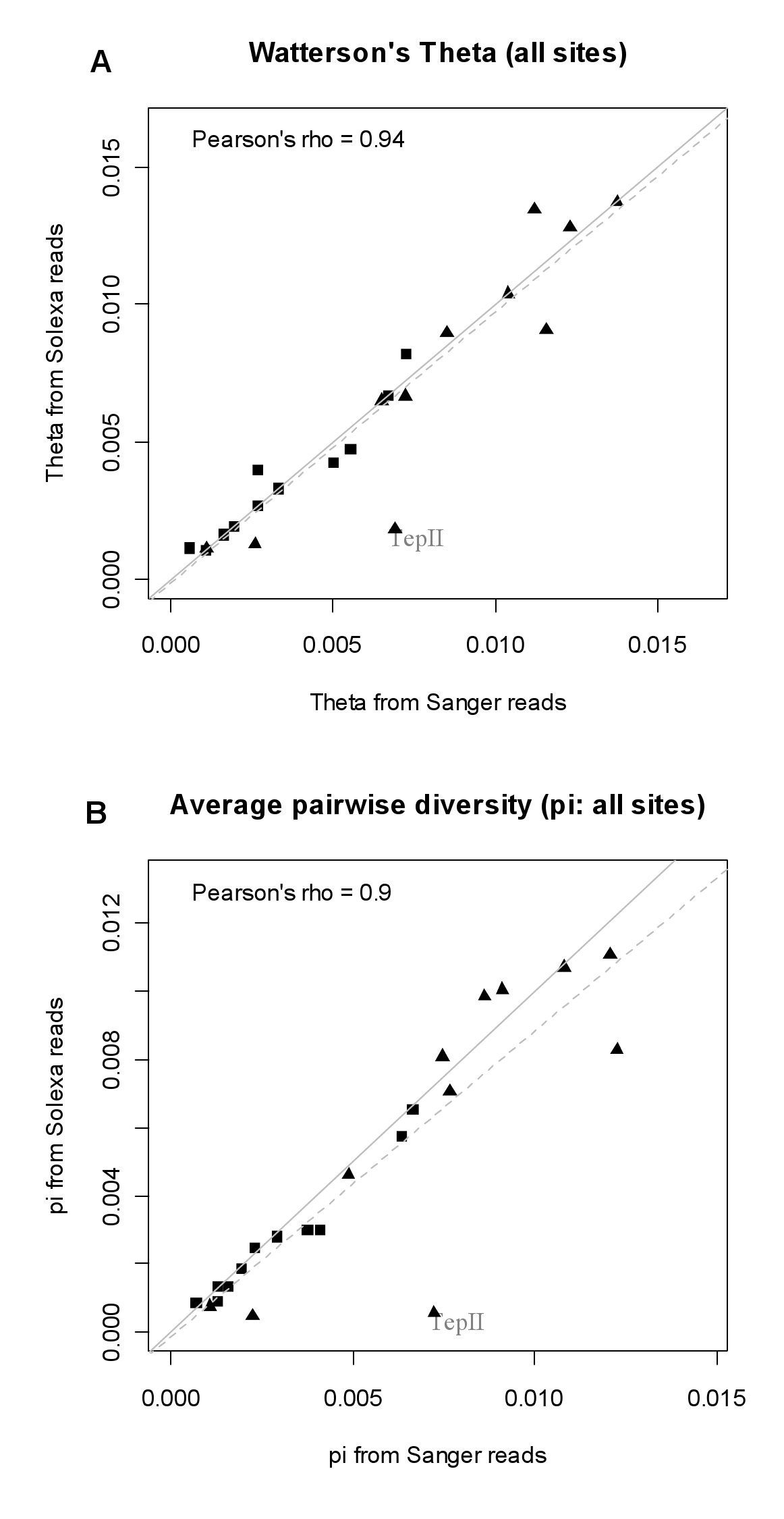

Supplement: Figure S8 — The relationship between Sanger and Solexa-Illumina estimates of diversity. To assess the impact of short-read sequencing errors on estimates of diversity we re-sequenced 11 loci in the Greek (Athens) populations of D. melanogaster and D. simulans (see Text S1 supplementary methods). (A) shows the relationship between θw estimated from Sanger sequences and from θw estimated from short-read sequences, (B) shows the same for average pairwise diversity (θπ). Triangles are loci re-sequenced in D. simulans and squares are loci re-sequenced in D. melanogaster; the solid lines depict a 1∶1 relationship, and dashed lines a linear regression of short-read estimates on Sanger estimates. Much of the difference between the two estimates is due to allelic dropout in D. simulans TepII, caused by a segregating indel at the site of the Solexa Long-PCR primer. (0.17 MB TIF) [file pgen.1000698.s008.tif]

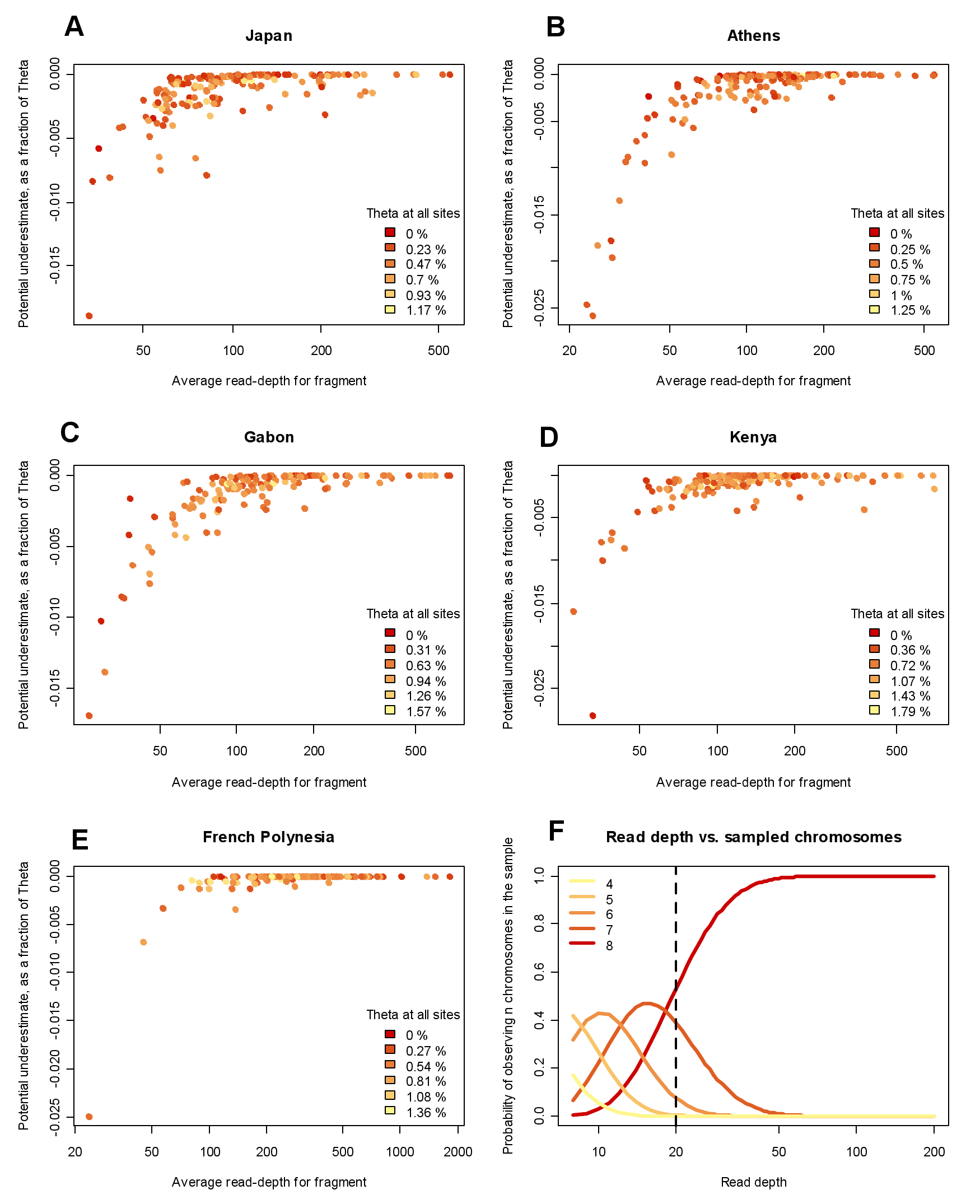

Supplement: Figure S9 — Underestimates of Watterson's θw due to un-sampled genomes. In calculating θw we assumed that all 8 chromosomes were sampled. However, at low coverage sites (<50-fold) it is unlikely that this is the case, and this could potentially lead to underestimates of θw. We have calculated the effect of this on our estimates under the assumption that all the chromosomes are equally represented in the template pool and are sampled at random in the short reads. We find that the effect is small (A–E, below). Given our read depths for each locus in each population, we underestimate θw by <3% of the correct value at very low coverage and <0.5% at most loci. This is because (F) even at 20-fold coverage there is >90% chance of sampling 7 or 8 chromosomes, and the denominator of Wattersons's estimator (Σn-1 i = 1(1/i)) differs little between n = 7 and n = 8. (0.35 MB TIF) [file pgen.1000698.s009.tif]

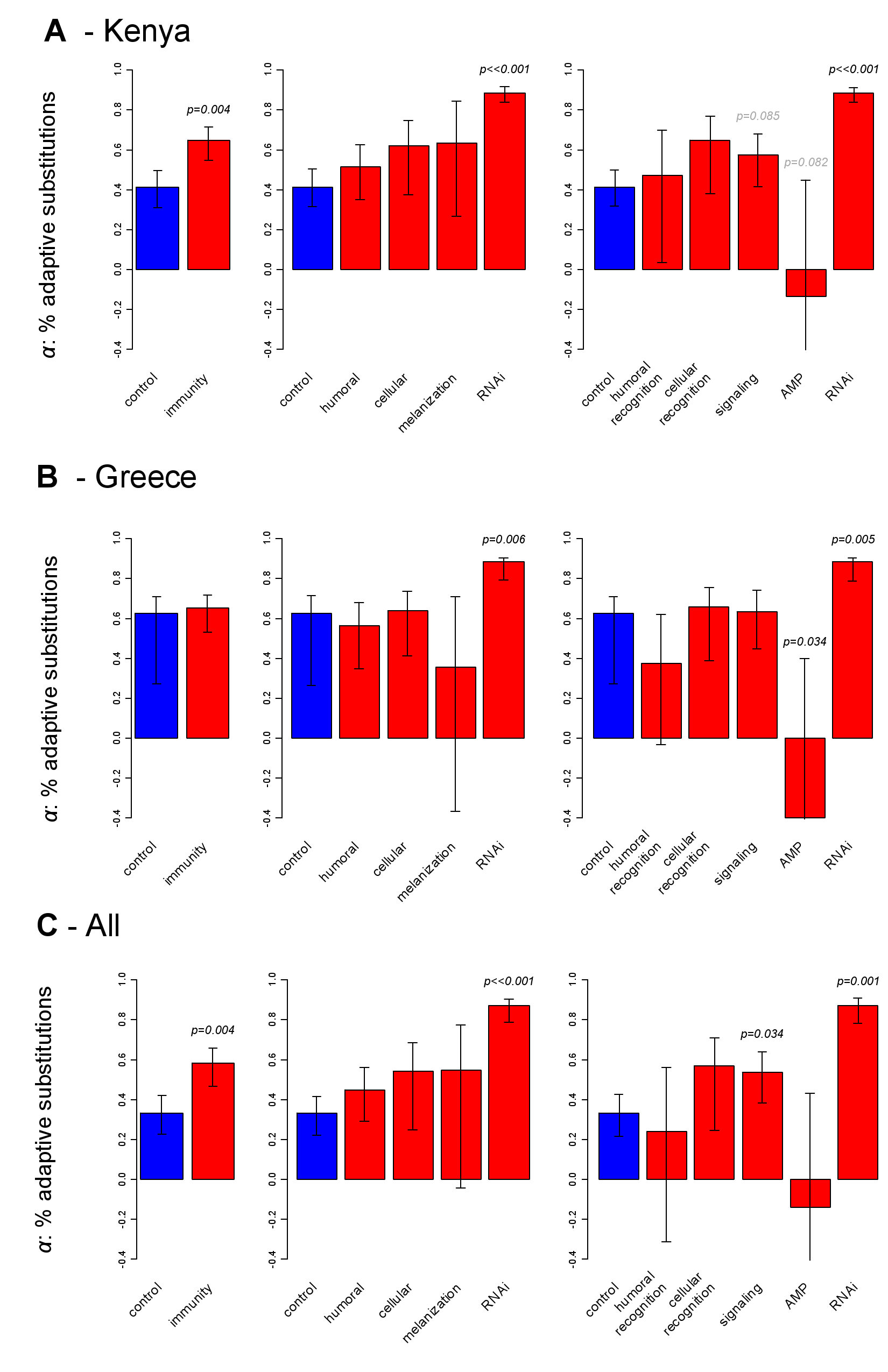

Supplement: Figure S10 — The estimated proportion of adaptive substitutions inferred by using polymorphism data from different populations. Graphs show the estimated proportion of amino acid substitutions fixed by selection (α) between D. melanogaster and D. simulans using data from different populations. (A) Kenyan populations only, based on 8 chromosomes of each (reproduced from the main text for comparison); (B) Greek populations only, based on 8 chromosomes of each; (C) All 8 populations (6 D. melanogaster and 2 D. simulans), based on 48 chromosomes of D. melanogaster and 16 chromosomes of D. simulans. Note that absolute estimates are smaller when all populations are used in the analysis, probably due to more rare variants. Error bars are 95% bootstrap intervals from re-sampling genes within classes; p-values are relative to the “control” genes, assessed by bootstrapping. (0.27 MB TIF) [file pgen.1000698.s010.tif]

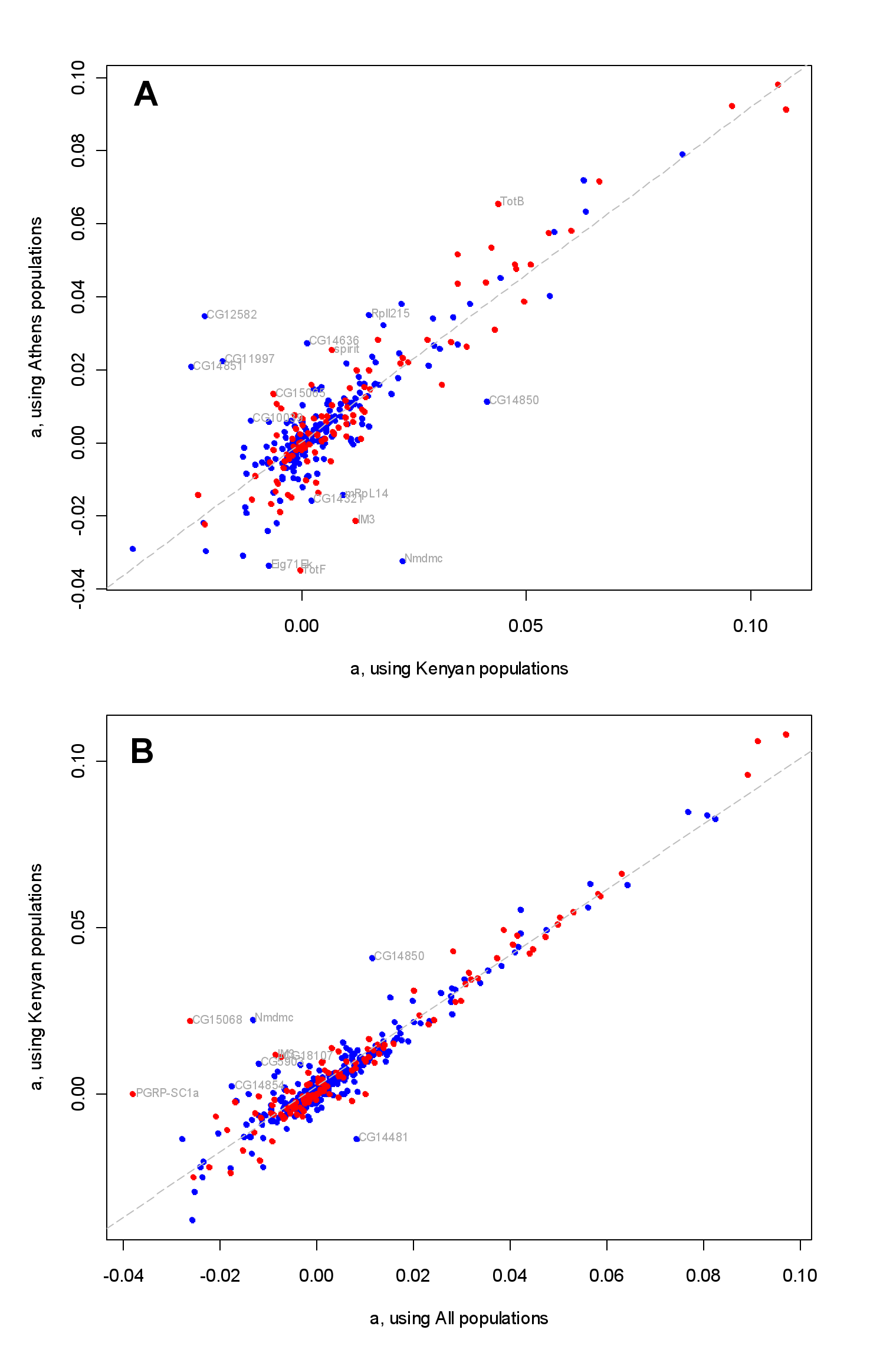

Supplement: Figure S11 — The correlation between estimates of a from different sample populations. The estimated number of adaptive substitutions per non-synonymous site (a) was little affected by the choice of population to provide polymorphism data. (A) shows the correlation in a between estimates using single Greek populations of D. melanogaster and D. simulans, and estimates using single Africa populations of D. melanogaster and D. simulans (both using D. melanogaster-D. simulans divergence) (Pearson's product-moment correlation = 0.87, p<2×10^−16^). (B) shows the correlation between a single African population and all combined populations of both species (Pearson's product-moment correlation = 0.95, p<2×10^−16^). Thus estimates of a are similar when using African and non-African populations, and small African samples (8 chromosomes per species) provide almost the same information as global samples (48 D. melanogaster chromosomes and 16 D. simulans chromosomes). Immune genes are shown in red, and other genes in blue. Visually identified outliers are labelled. (0.20 MB TIF) [file pgen.1000698.s011.tif]

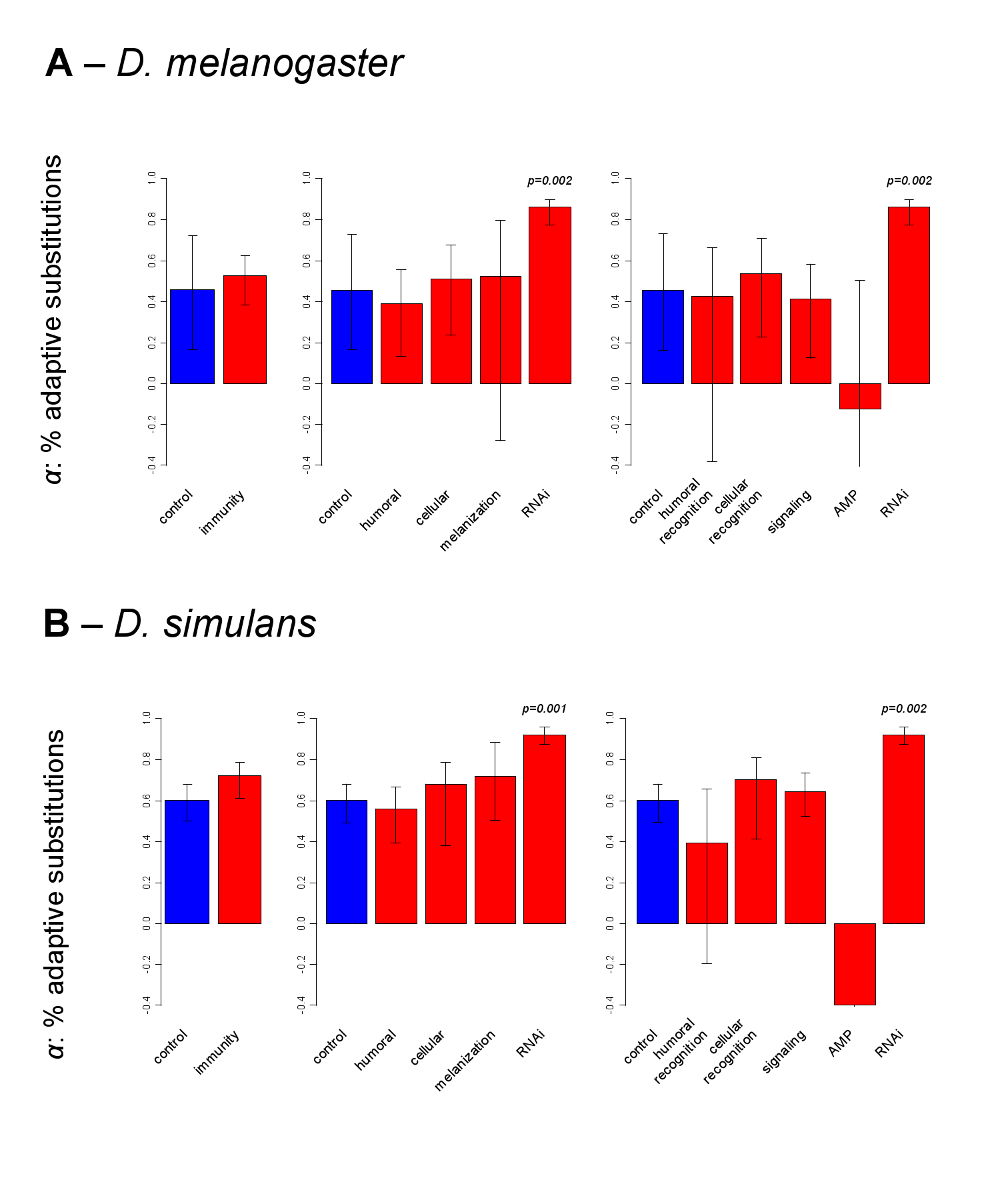

Supplement: Figure S12 — The estimated proportion of adaptive substitutions inferred separately along the D. melanogaster and D. simulans lineages using Kenyan populations. By using D. yakuba and D. erecta to infer the state of the D. melanogaster-D. simulans common ancestor, substitutions can be assigned to the D. melanogaster or D. simulans lineage alone, and α inferred for each species separately. (A) D. melanogaster using a single Kenyan population only; (B) D. simulans using a single Kenyan population only. Note that immunity and control genes do not differ significantly, but this is probably due to the low power associated with the very small divergence. Interestingly, although the pattern across gene classes is qualitatively identical between species, absolute estimates of α are consistently higher in D. simulans. Error bars are 95% bootstrap intervals from re-sampling genes within classes, and p-values are relative to the control genes, assessed by bootstrapping. (0.17 MB TIF) [file pgen.1000698.s012.tif]

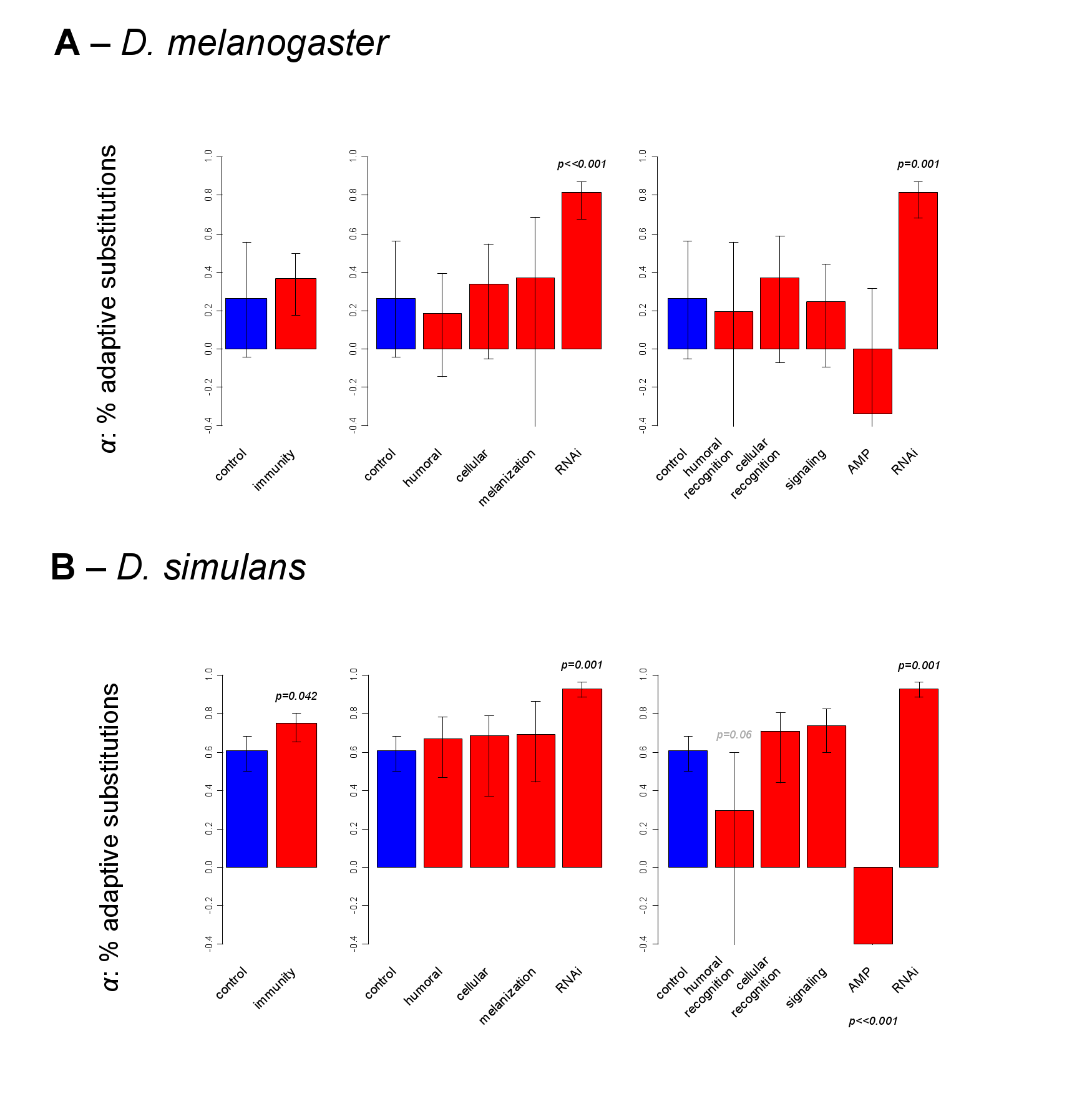

Supplement: Figure S13 — The estimated proportion of adaptive substitutions inferred separately along the D. melanogaster and D. simulans lineages using all sampled populations. By using D. yakuba and D. erecta to infer the ancestral state of the D. melanogaster–D. simulans common ancestor, α was inferred separately for each species (see Figure S12 above). (A) D. melanogaster using polymorphism data from all six D. melanogaster populations; (B) D. simulans using both Kenyan and Athens populations. As in Figure S12, the immunity-control comparison is not significant for D. melanogaster, and estimates of α are consistently much higher in D. simulans. However, unlike Figure S12, here the effect of species is conflated with the number of sampled populations, and thus the presence of rarer alleles in D. melanogaster. Error bars are 95% bootstrap intervals from re-sampling genes within classes, and p-values are relative to the control genes, assessed by bootstrapping. (0.17 MB TIF) [file pgen.1000698.s013.tif]

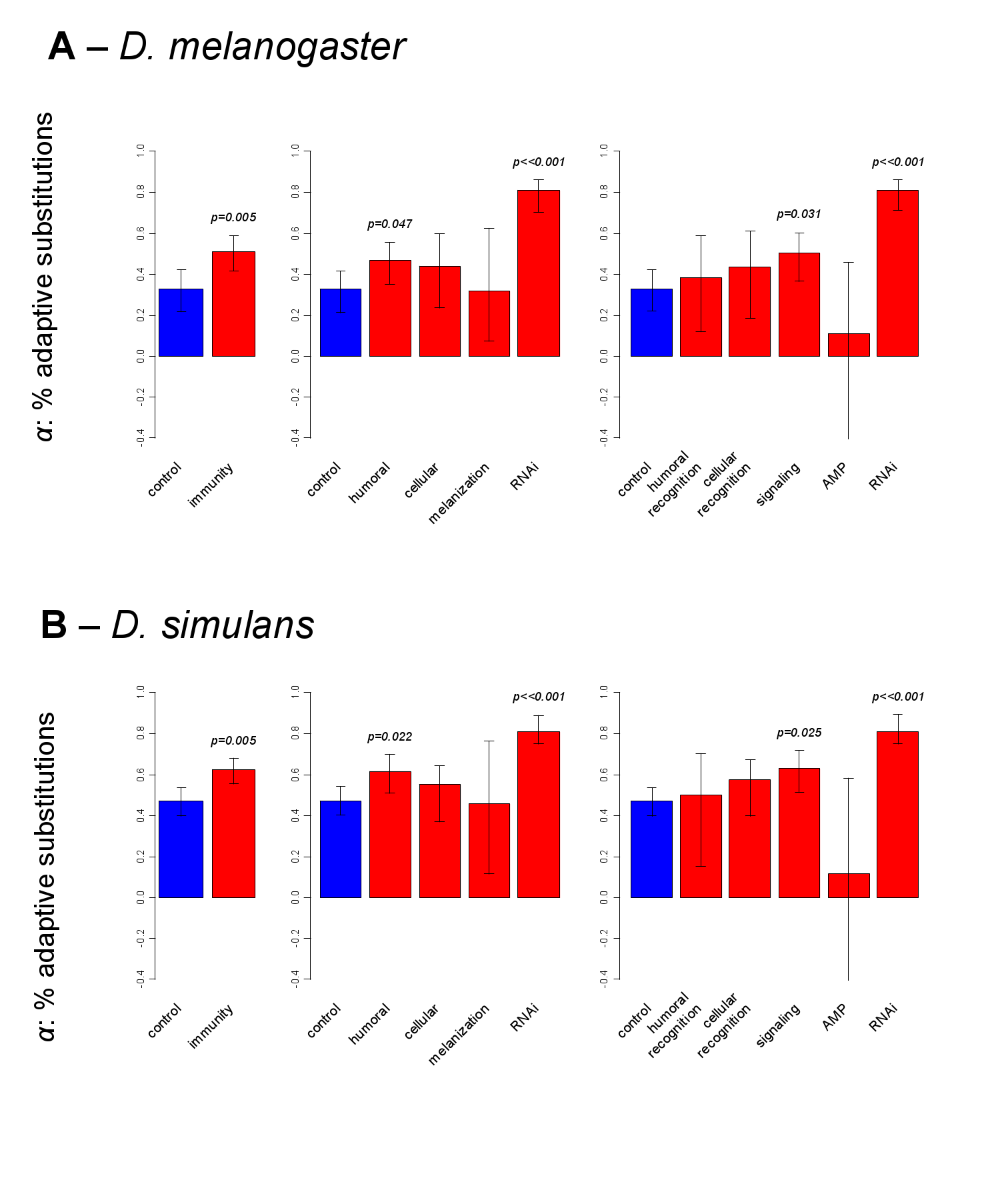

Supplement: Figure S14 — The estimated proportion of adaptive substitutions between D. yakuba and D. melanogaster/simulans using Kenyan populations. (A) D. melanogaster vs D. yakuba, using a single Kenyan population of D. melanogaster; (B) D. simulans vs. D. yakuba using a single Kenyan population D. simulans. Although the pattern across gene classes is qualitatively identical between species, absolute estimates of α are consistently higher in D. simulans (see also Figures S12, S13). Error bars are 95% bootstrap intervals from re-sampling genes within classes, and p-values are relative to the control genes, assessed by bootstrapping. (0.17 MB TIF) [file pgen.1000698.s014.tif]

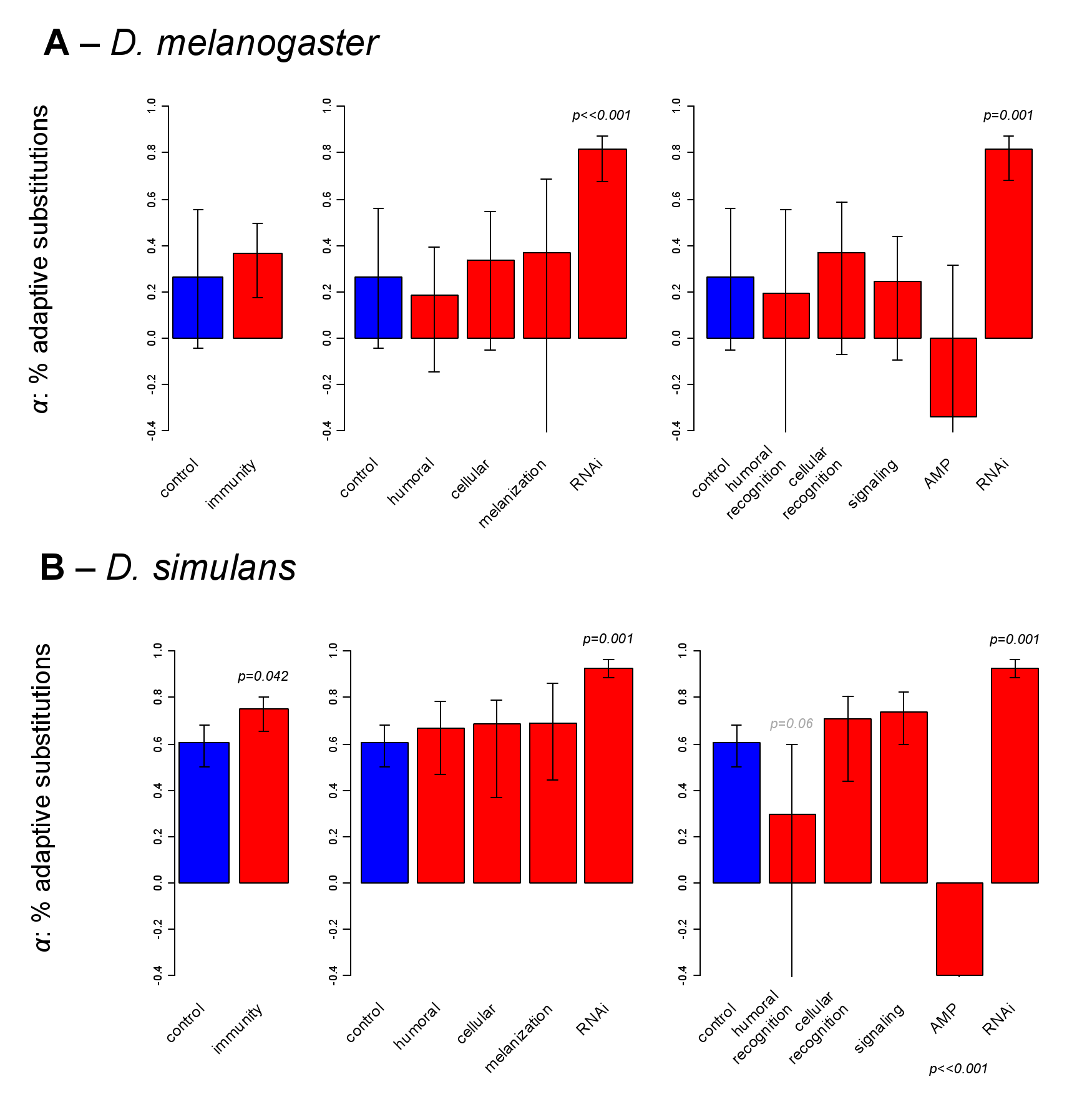

Supplement: Figure S15 — The estimated proportion of adaptive substitutions between D. yakuba and D. melanogaster/simulans using all sampled populations. (A) D. melanogaster vs D. yakuba, using all sampled D. melanogaster populations; (B) D. simulans vs. D. yakuba using both D. simulans populations. As in Figures S12, S13, S14, absolute estimates of α are consistently much higher in D. simulans, however, unlike Figure S14, here the effect of species is conflated with the number of sampled populations, and thus the presence of rarer alleles in D. melanogaster. Error bars are 95% bootstrap intervals from re-sampling genes within classes, and p-values are relative to the control genes, assessed by bootstrapping. (0.20 MB TIF) [file pgen.1000698.s015.tif]

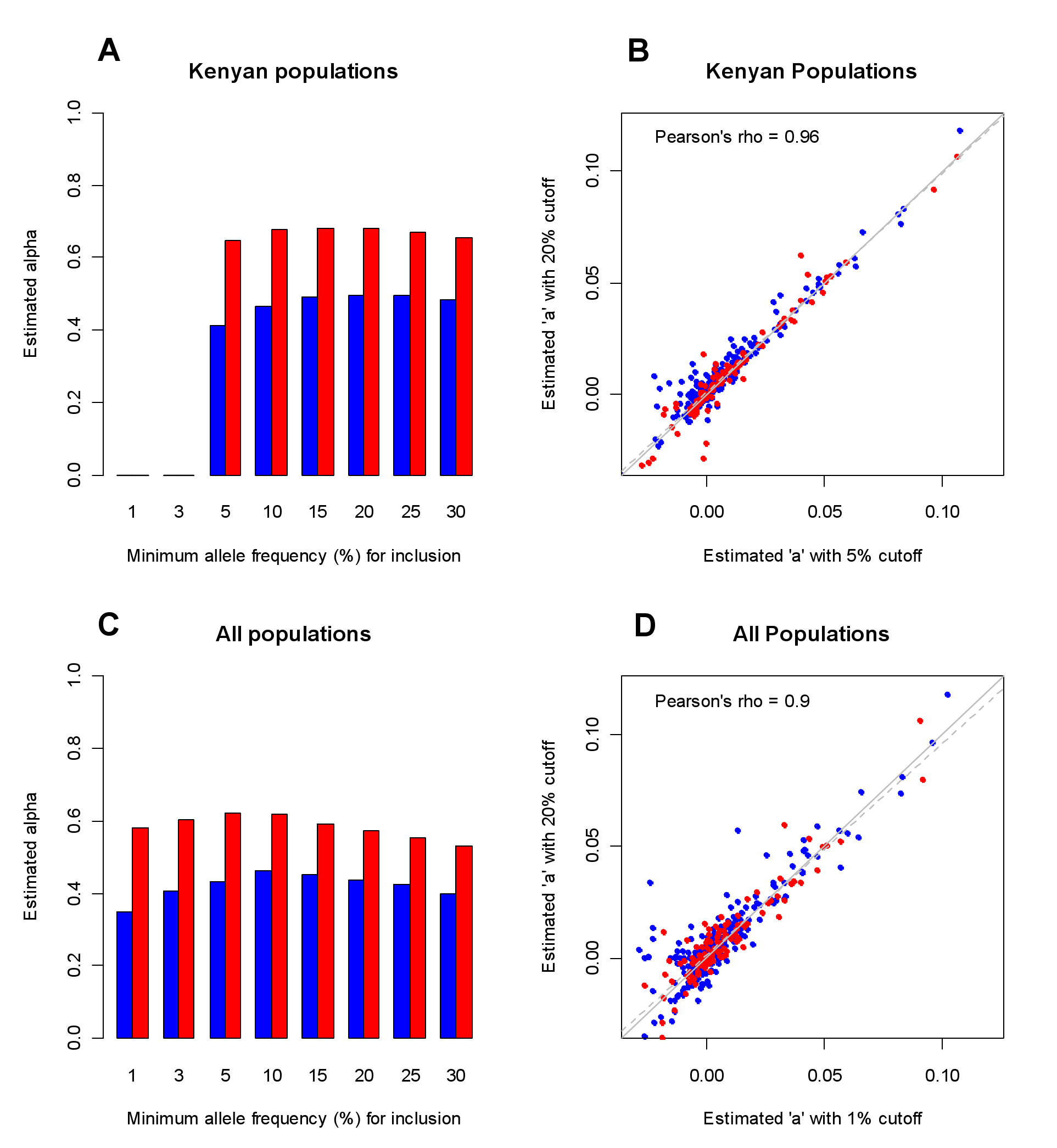

Supplement: Figure S16 — The estimated proportion of adaptive substitutions inferred by using polymorphism data from alleles that appear at different frequencies. Graphs show the estimated amount of adaptive substitution between D. melanogaster and D. simulans, based on polymorphism data from both species, for Kenyan populations only (A and B) and for all populations (C and D). (A and C) show the effect of excluding low-frequency alleles on α (the estimated proportion of adaptive substitutions) for classes of immune (red) and non-immune (blue) genes. Note there is a 5% frequency threshold per-population for inclusion in any of the analyses (See main text). (B and D) show the effect of excluding low-frequency alleles on a (the estimated number of adaptive substitutions per non-synonymous site) for immune (red) and non-immune (blue) genes individually. The solid grey line represents a 1∶1 correspondence, the dashed line a linear regression. The effect of excluding rare variants is both small, and consistent with theoretical expectations. This suggests that the presence of weakly-deleterious alleles that slightly depress estimates of α, but do not have a substantial impact upon our conclusions. It also suggests that our sequencing errors and inclusion-threshold have a minimal impact upon our conclusions. (0.28 MB TIF) [file pgen.1000698.s016.tif]

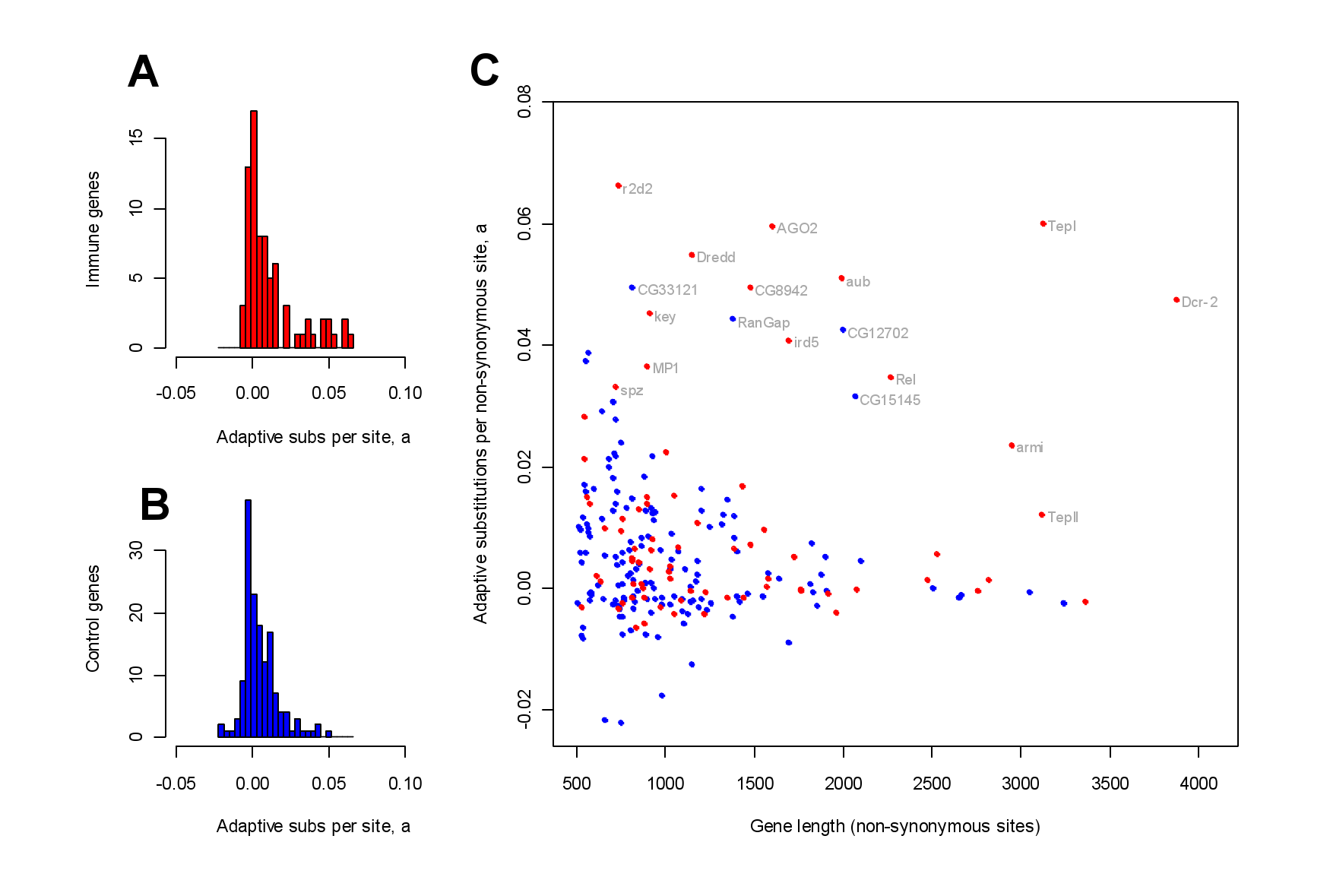

Supplement: Figure S17 — The distribution of the number of adaptive substitutions (a) between genes, excluding short genes. Although mean a (the number of adaptive substitutions per non-synonymous site) is significantly higher for immune genes than for other genes, the modal class is similar and the variance larger (see main text). The greater variance in non-immunity genes could be attributed to shorter sampled gene length giving rise to greater sampling error. However, the exclusion of short genes from both classes does not alter the effect, as variance in immunity genes is still greater than that in non immunity genes (A–C); Var(a)×10−4 = 3.2 vs. 1.3, p = 0.0017. Immune genes are shown in red, and other genes in blue. Note that we used a in place of α for this per-gene analysis because α is poorly estimated for single genes (see Methods). (0.12 MB TIF) [file pgen.1000698.s017.tif]

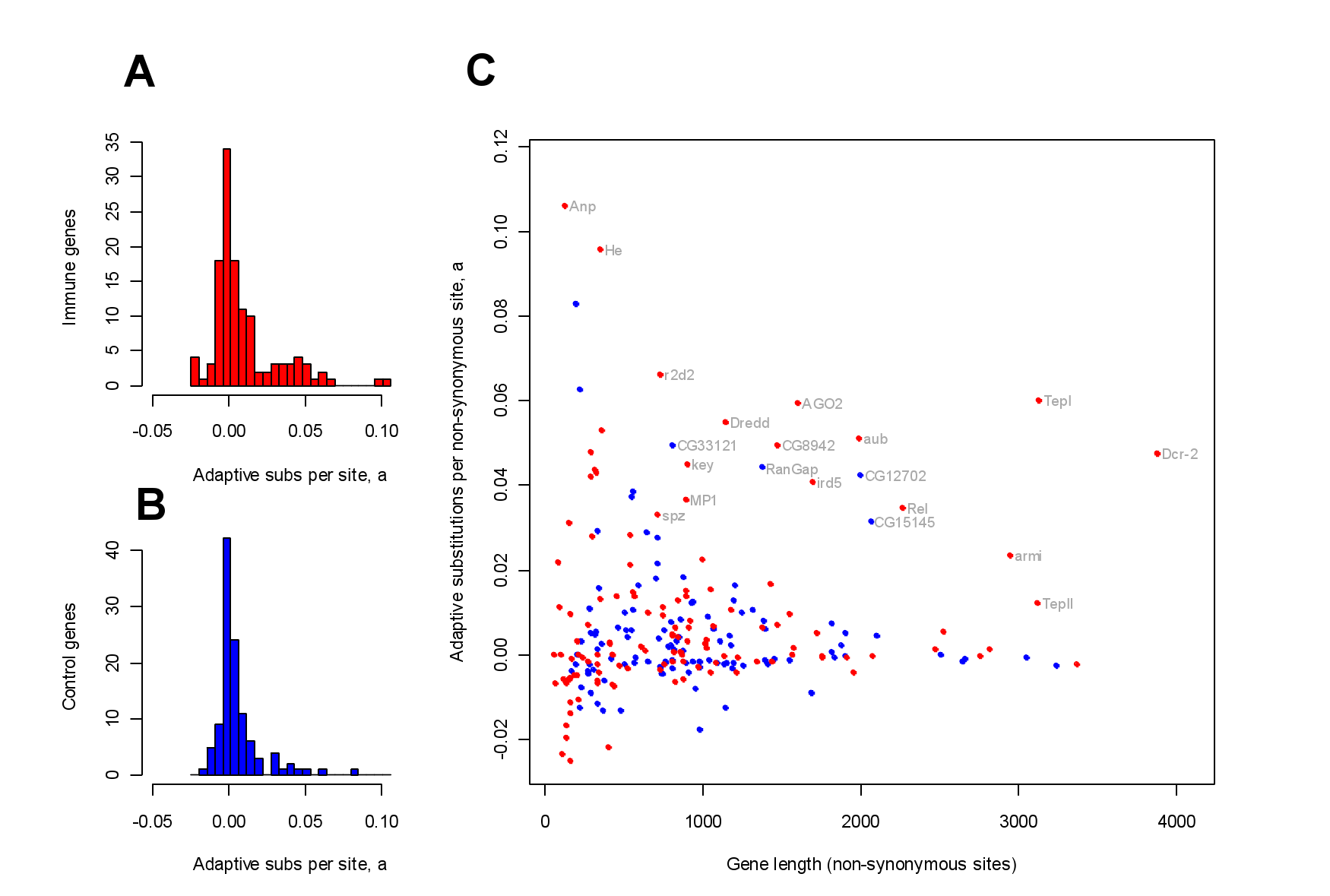

Supplement: Figure S18 — The distribution of the number of adaptive substitutions (a) between genes, using only genes intentionally targeted by PCR. The greater variance in non-immunity genes (see Figure S17) might also be attributed to shorter gene length giving rise to greater sampling error. In our primary dataset there are a large number of short gene fragments from non-immunity genes that appear in our sample merely because they happened to occur within the amplicons of a “targeted” gene. However, the exclusion of these “un-targeted” genes does not alter the effect. Variance in immunity genes is still greater than non immunity genes (A–C); Var(a)×10−4 = 4.8 vs. 2.3, p = 0.0177. Immune genes are shown in red, and other genes in blue. (0.12 MB TIF) [file pgen.1000698.s018.tif]

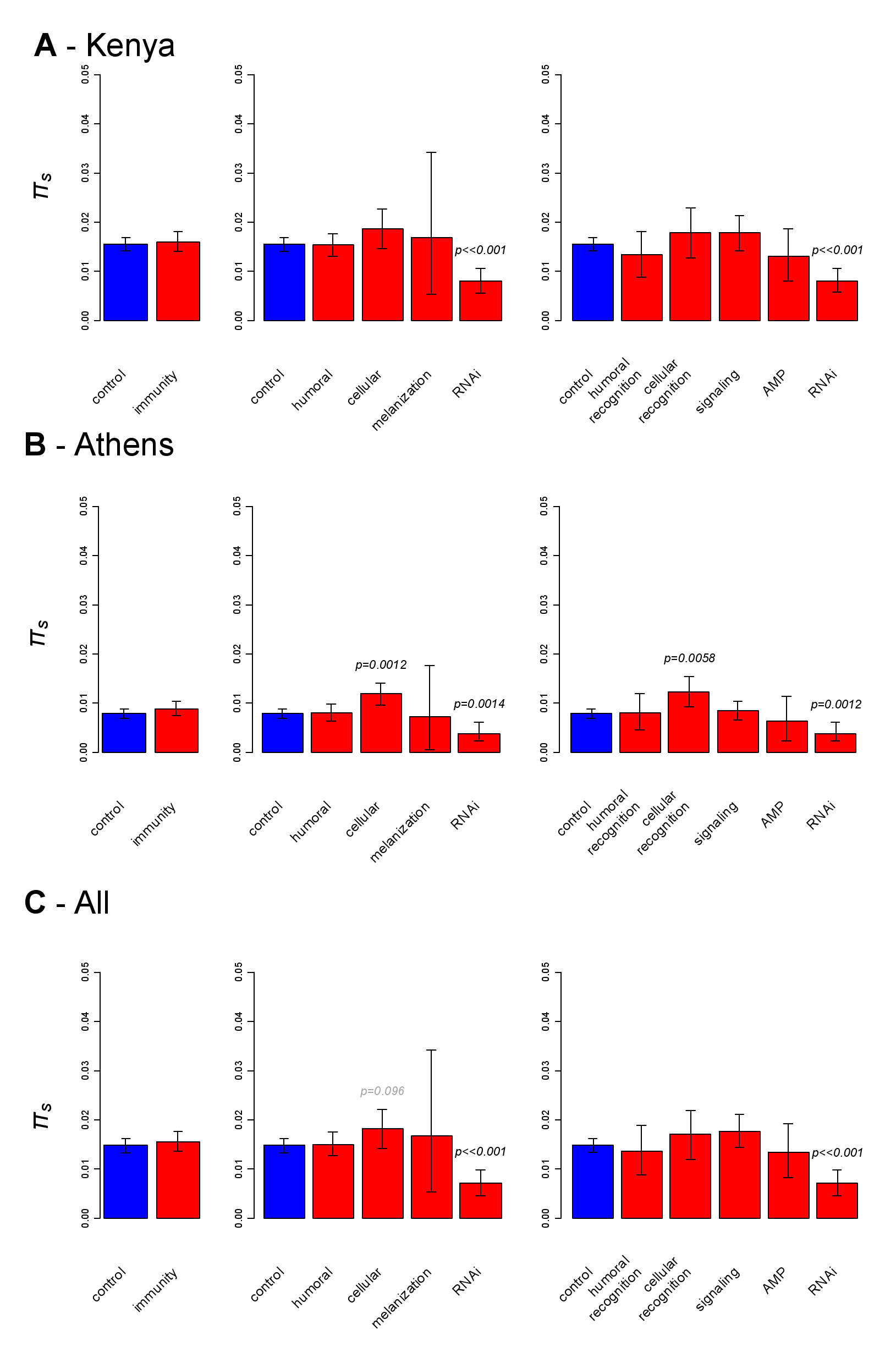

Supplement: Figure S19 — Neutral diversity in D. melanogaster. Genetic diversity at synonymous sites in immunity and non-immunity genes (πs). Note that we do not have direct estimates of allele frequency (see Methods), and instead we use read frequency as a surrogate to calculate π. However, results based on Watterson's θ were very similar, and our estimates of πs and θw were very highly correlated (r2>0.95 in each population). (A) Kenya; (B) Athens; (C) All D. melanogaster populations combined. Error bars are 95% bootstrap intervals of the mean from re-sampling genes within classes, and p-values are relative to the control genes, assessed by bootstrapping. (0.22 MB TIF) [file pgen.1000698.s019.tif]

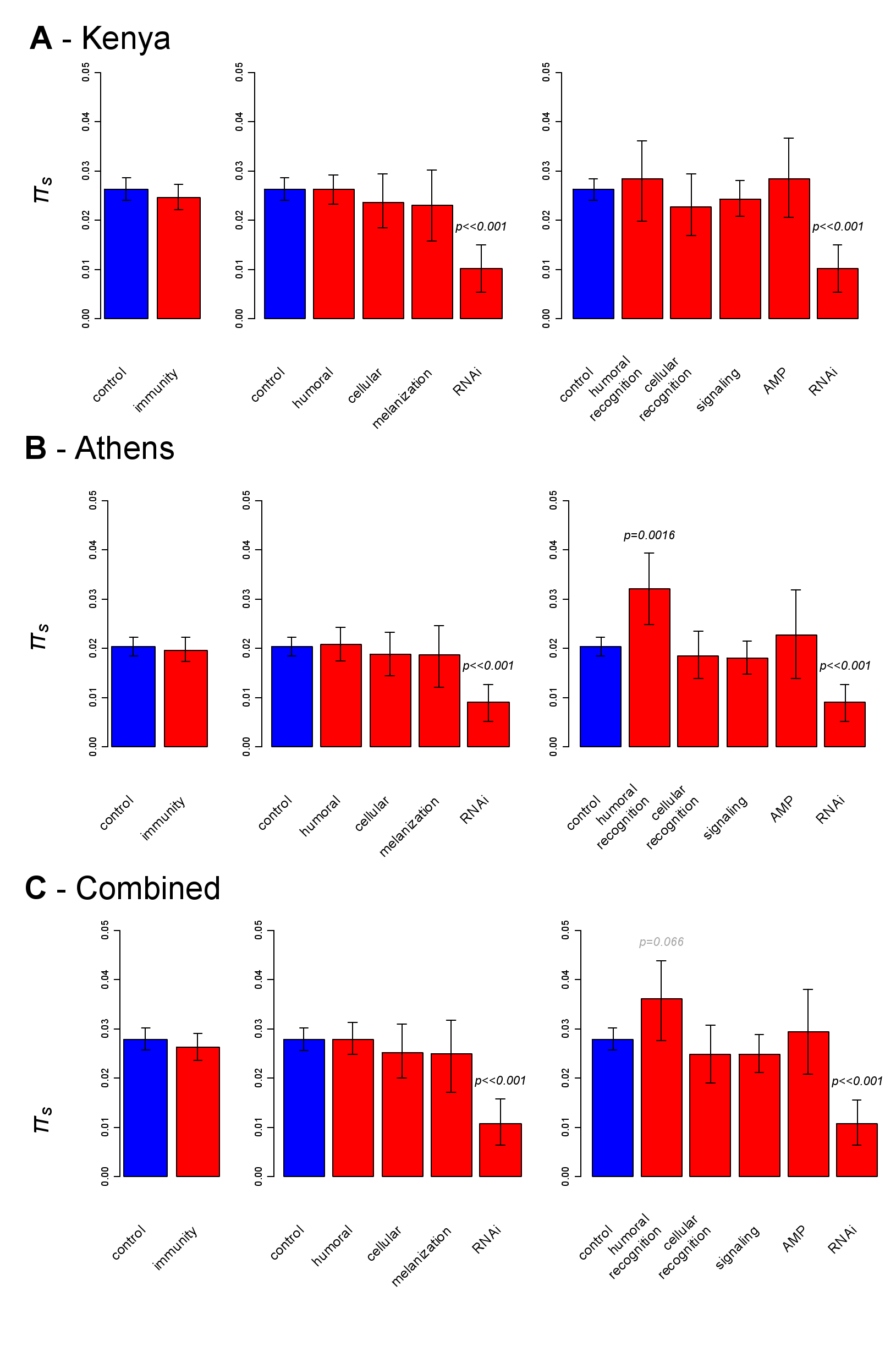

Supplement: Figure S20 — Neutral diversity in D. simulans. Genetic diversity at synonymous sites in immunity and non-immunity genes (πs). (see Figure S19 for details). Again, results based on Watterson's θ were very similar, as our estimates of πs and θw were very highly correlated (r2>0.93 in each population). (A) Kenya; (B) Athens; (C) Both populations combined. Error bars are 95% bootstrap intervals of the mean from re-sampling genes within classes, and p-values are relative to the control genes, assessed by bootstrapping. (0.24 MB TIF) [file pgen.1000698.s020.tif]

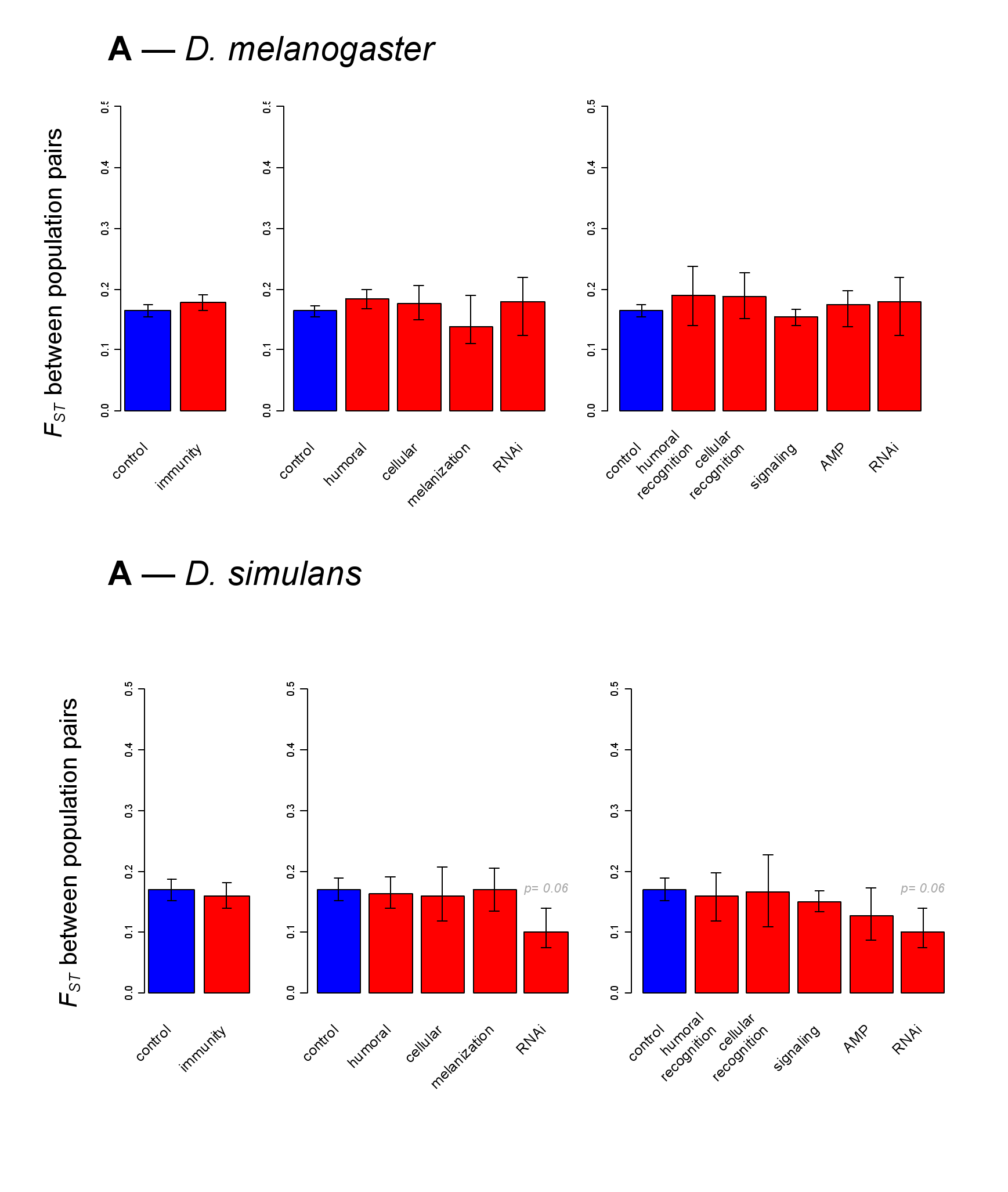

Supplement: Figure S21 — Genetic differentiation (FST) between populations. Genetic differentiation between populations (FST) at synonymous-sites in immunity and non-immunity genes. Error bars are 95% bootstrap intervals of the mean from re-sampling genes within classes, and p-values are relative to the control genes, assessed by bootstrapping. (0.18 MB TIF) [file pgen.1000698.s021.tif]

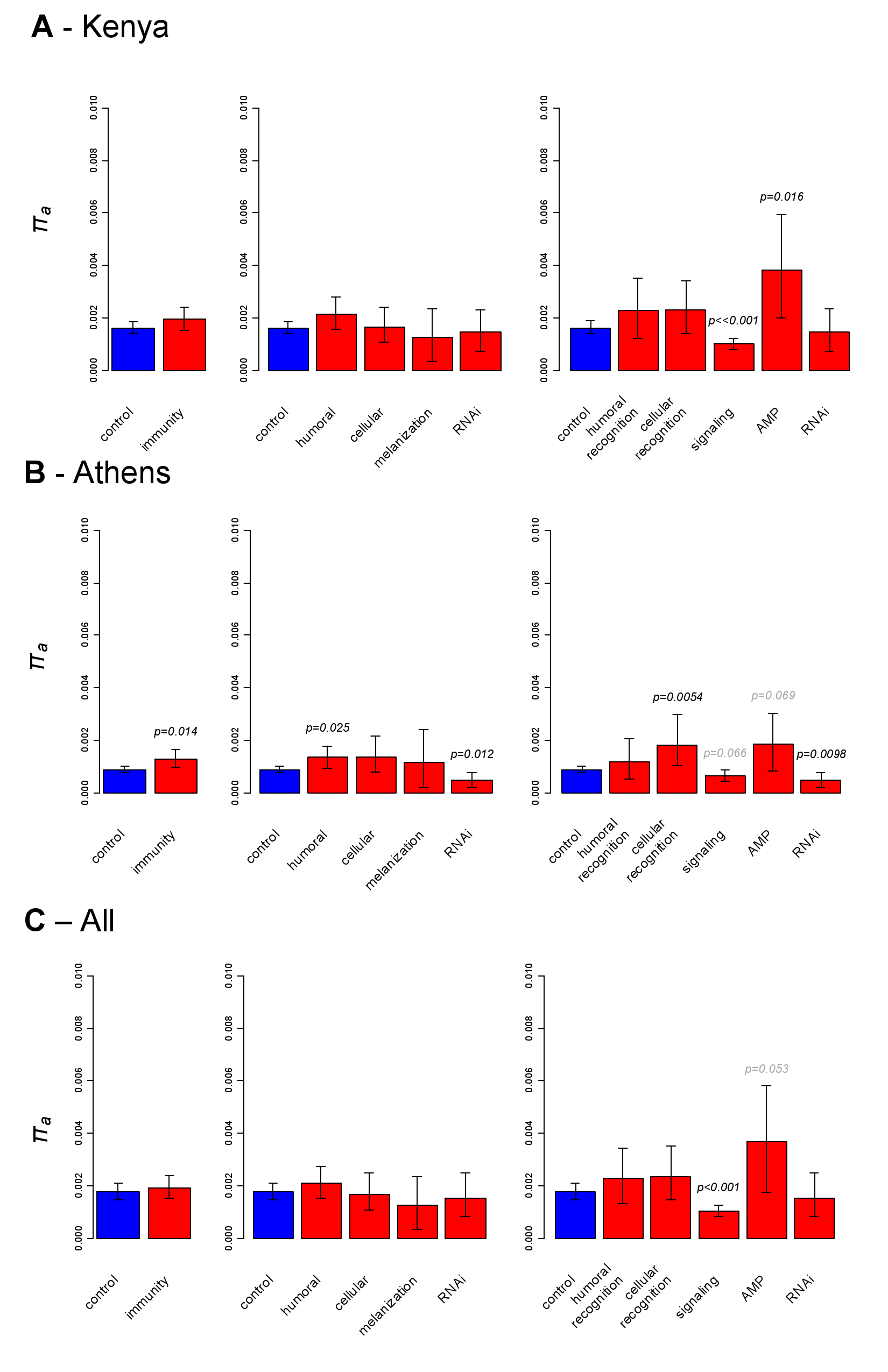

Supplement: Figure S22 — Non-synonymous diversity in D. melanogaster. Genetic diversity at non-synonymous-sites in immunity and non-immunity genes (πa). (A) Kenya; (B) Athens; (C) All D. melanogaster populations combined. Error bars are 95% bootstrap intervals of the mean from re-sampling genes within classes, and p-values are relative to the control genes, assessed by bootstrapping. (0.22 MB TIF) [file pgen.1000698.s022.tif]

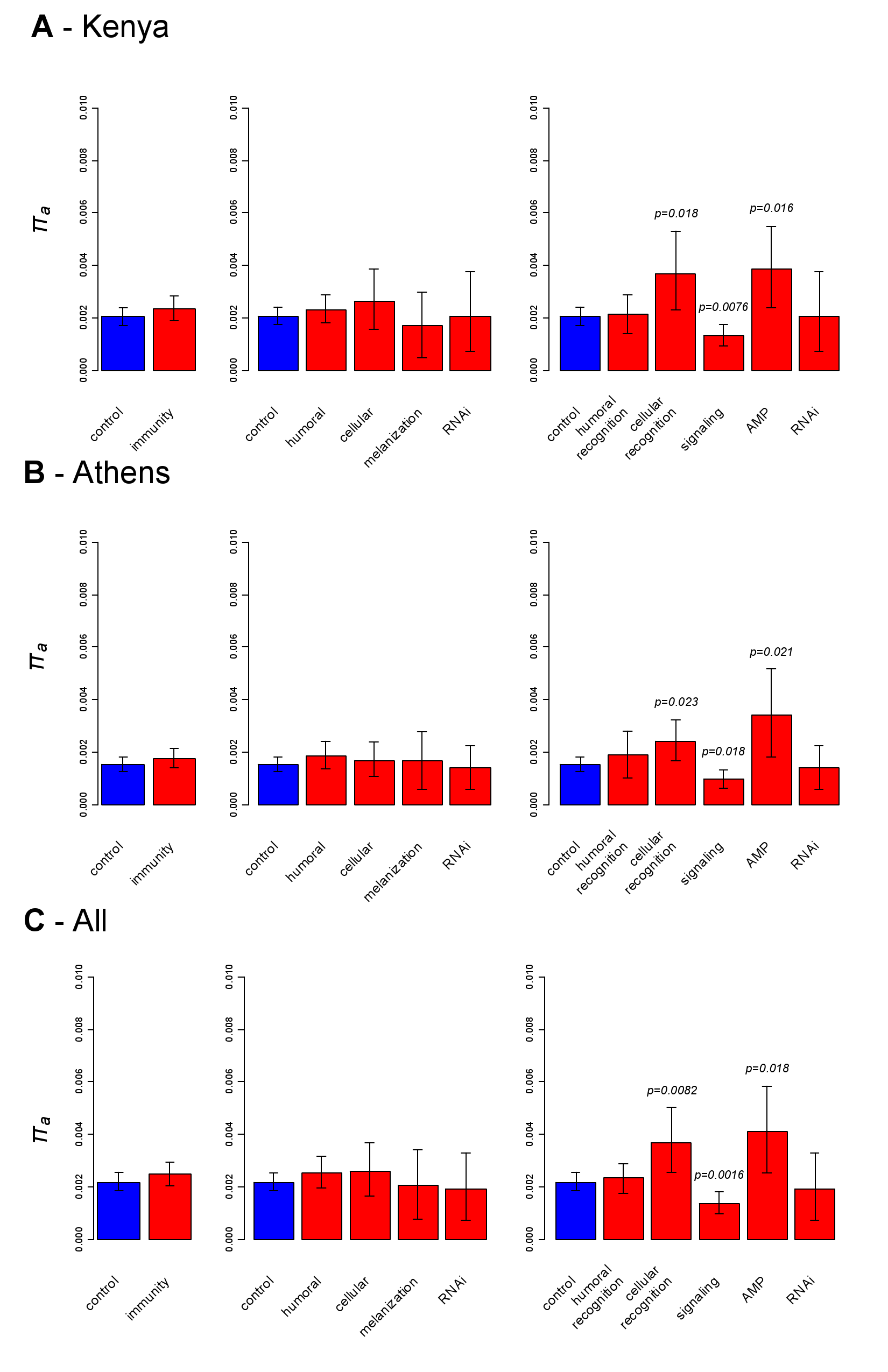

Supplement: Figure S23 — Non-synonymous diversity in D. simulans. Genetic diversity at non-synonymous-sites in immunity and non-immunity genes (πa). (A) Kenya; (B) Athens; (C) Both populations combined. Error bars are 95% bootstrap intervals from re-sampling genes within classes, and p-values are relative to the control genes, assessed by bootstrapping. (0.23 MB TIF) [file pgen.1000698.s023.tif]

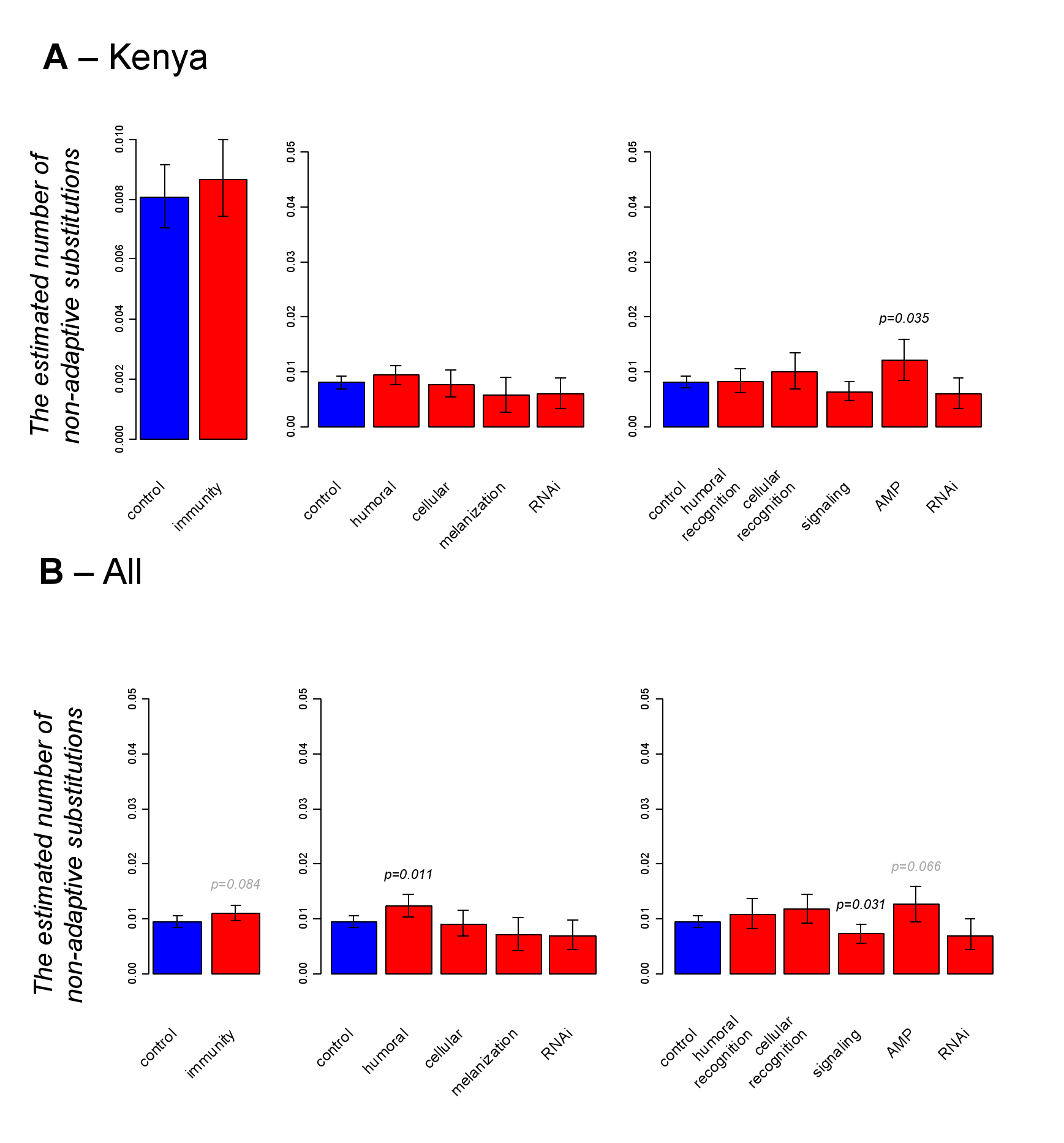

Supplement: Figure S24 — The estimated number of non-adaptive substitutions per site between D. melanogaster and D. simulans. The estimated number of substitutions per non-synonymous site that were driven by genetic drift is shown. This number was estimated from (Dn/Ln)-a, where a is the estimated number of adaptively-driven substitutions; note that when a is separately parameterized at each locus, this removes from the estimates any dependency on the observed Dn values. The estimates of drift-mediated substitutions are less variable within categories of locus than are estimates of adaptive substitution (although this must be partly due to the lack of dependence on the observed Dn decreasing error variance). There are also fewer significant differences between classes of locus, notably a lack of difference between immunity and control genes. (A) Kenyan populations only; (B) All 8 populations (6 D. melanogaster and 2 D. simulans). Error bars are 95% bootstrap intervals from re-sampling genes within classes, and p-values are relative to the control genes, assessed by bootstrapping. (0.18 MB TIF) [file pgen.1000698.s024.tif]

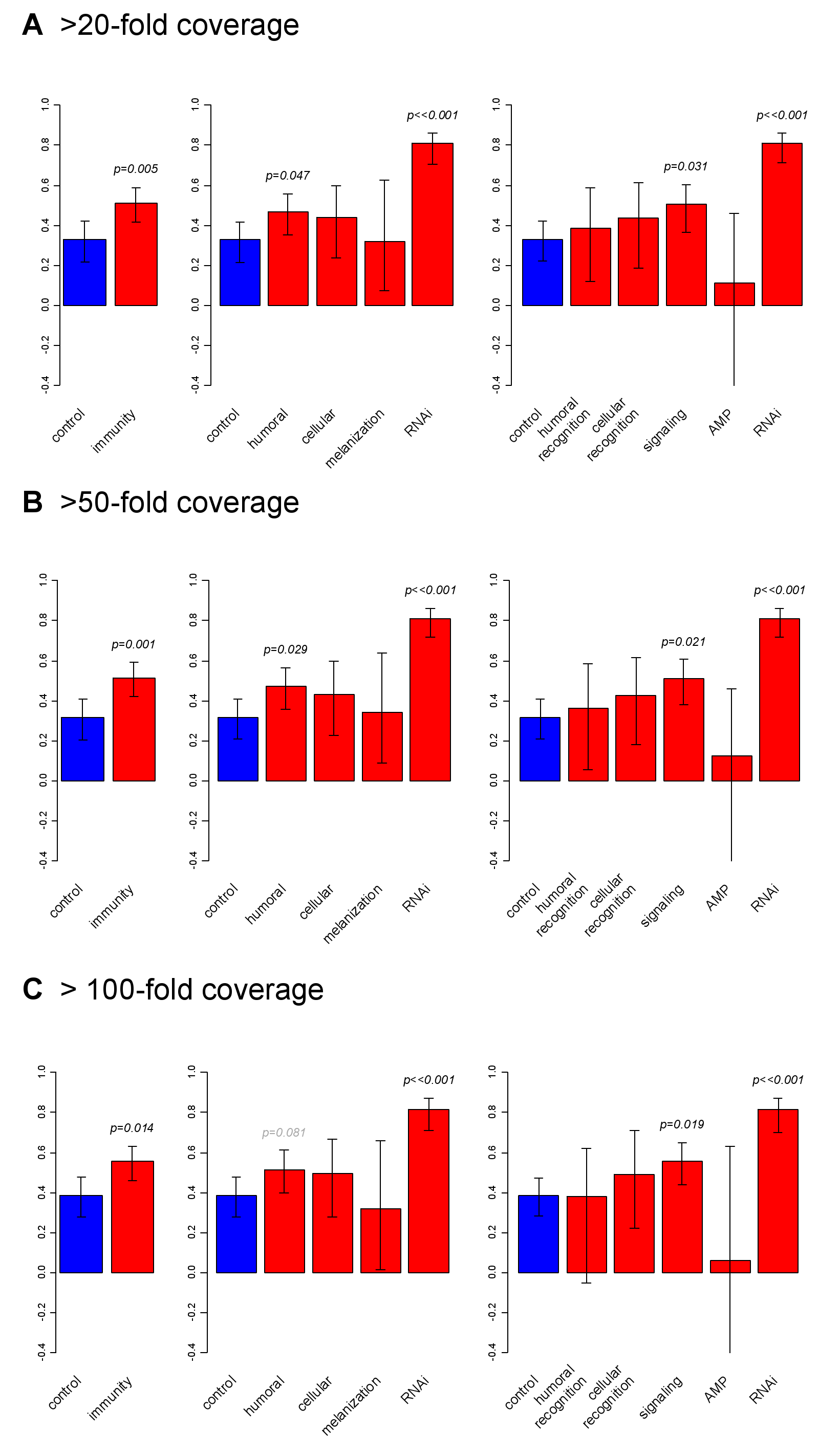

Supplement: Figure S25 — The estimated proportion of adaptive substitutions (α) between D. melanogaster (Kenya population) and D. yakuba according to read depth. Limiting the analysis to sites of high depth of coverage (>50-fold, >100-fold) has little impact on inferred rates of adaptive evolution. (0.28 MB TIF) [file pgen.1000698.s025.tif]

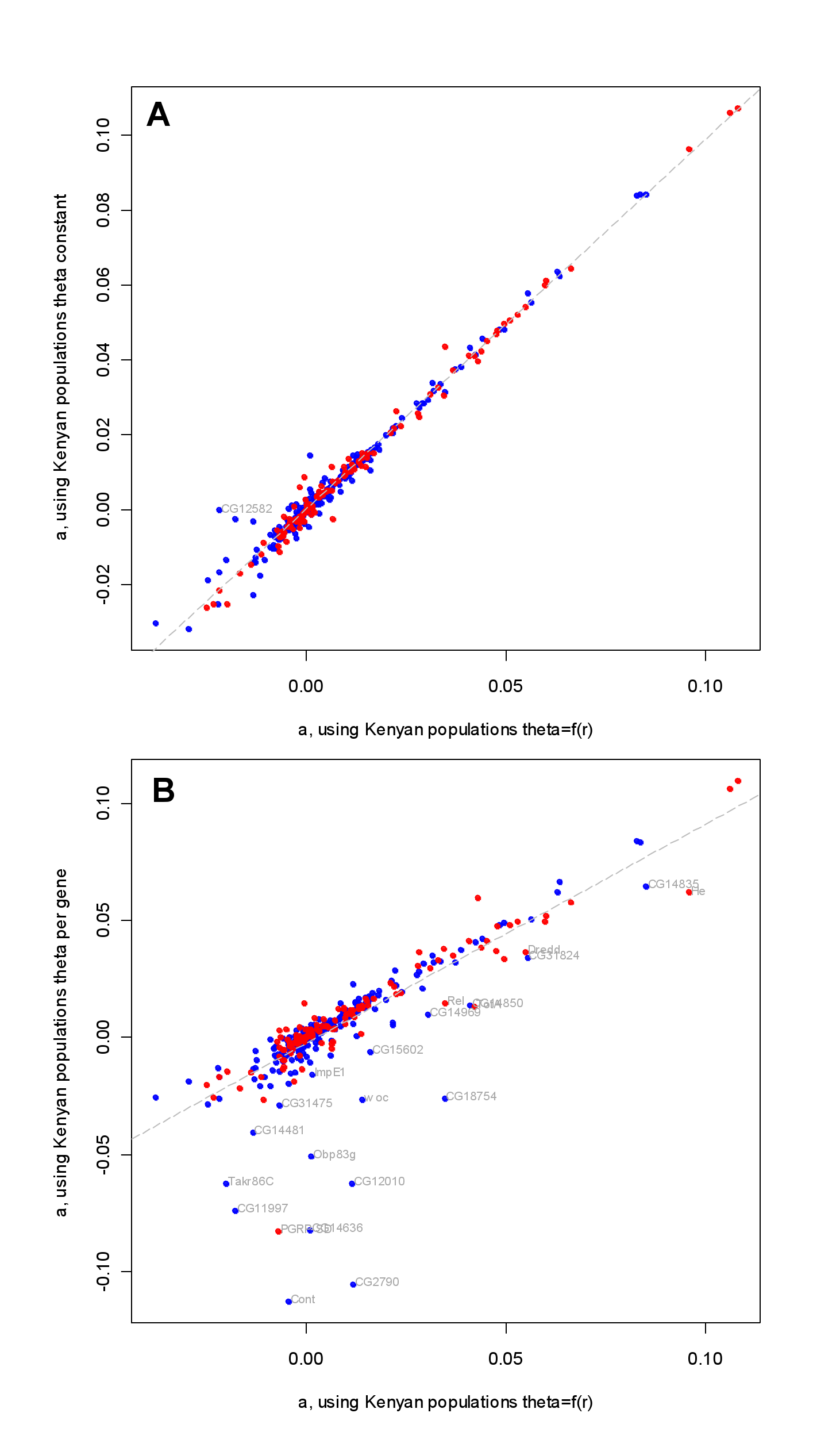

Supplement: Figure S26 — Single-gene estimates of a using different models. To estimate single-gene a-values we fitted a model in which the parameter θ = 4Nμ was shared between loci as a linear function of recombination rate (see Methods). To explore the effect of this constraint, we compared our estimates of a to estimates derived using a single θ shared between all loci. (A) Pearson's correlation coefficient = 0.99, p<10–15), and separate estimates of θ for each locus. (B) Pearson's correlation coefficient = 0.75, p<10–15. In (B), the conspicuous outliers are almost all control genes that fell within the 5 Kbp amplicons, but which were not targets of primer design (see Text S1, detailed methods), and lack polymorphism data for D. simulans. This leads to over fitting at these loci when θ is a locus-specific parameter, and therefore poor estimation of a. In any case, use of the smaller model will tend to make our analyses conservative. (0.22 MB TIF) [file pgen.1000698.s026.tif]
