# Supplementary material for: Quantifying Adaptive Evolution in the Drosophila Immune System
Source: PLoS Genet. 2009 Oct 23;5(10):e1000698. doi: 10.1371/journal.pgen.1000698 (PMC2759075; doi:10.1371/journal.pgen.1000698)
Supplement: Table S2 — Model selection. The table gives parameters relevant to model-selection between different parameterizations of between-locus variation in α (the estimated proportion of amino-acid substitutions driven by positive natural selection). (2.70 MB PDF) [file pgen.1000698.s028.pdf]

Table S1: Model selection

The table gives parameters relevant to model-selection between different parameterizations of between-locus variation in α (the estimated proportion of amino-acid substitutions driven by positive natural selection).

α_i values	N^o. α_i	K	LnL	QAICc	w_i
All zero	0	400	-7461.7400	3433.6994	0.0000
Single value	1	401	-7083.0100	3311.1899	0.0000
Immunity and Control	2	402	-7022.6760	3294.1126	0.0000
Branch of immune system	6	406	-6917.8000	3271.0181	0.1977
Mode of action	7	407	-6900.5350	3268.2168	0.8023
Locus-specific	395	795	-6149.9648	4430.5030	0.0000

N^o. α_i: the total number of distinct α parameters in the model

K: total number of parameters

LnL: the maximised log likelihood

QAICc: The Akaike information criterion corrected for overdispersion, and finite sample size, QAICc = $-2\ln L/c + 2K + K(K+1)/(n-K-1)$. The sample size, n, was taken to be six times the number of loci = 2370, and the overdispersion correction, c, was twice the difference in log likelihoods for the largest model in the set, and the saturated model: $\ln L_{sat}$ = -3749.064, divided by the number of parameters in the largest model (K=795)

w: the Akaike weight, calculated as

$$w_i = \exp\left(-\tfrac{1}{2}\{\mathrm{QAIC}_i - \min(\mathrm{QAIC}_i)\}\right) \Big/ \sum_j \exp\left(-\tfrac{1}{2}\{\mathrm{QAIC}_j - \min(\mathrm{QAIC}_j)\}\right).$$
