# Supplementary material for: Quantifying Adaptive Evolution in the Drosophila Immune System
Source: PLoS Genet. 2009 Oct 23;5(10):e1000698. doi: 10.1371/journal.pgen.1000698 (PMC2759075; doi:10.1371/journal.pgen.1000698)
Supplement: Table S3 — Synonymous and non-synonymous diversity. Synonymous and non-synonymous diversity for different categories of gene in all populations, with those categories that were individually significantly different (p<0.05) from the control genes highlighted. (2.41 MB PDF) [file pgen.1000698.s029.pdf]

Table S2: Synonymous and Non-synonymous diversity for different categories of gene in all populations
(red text indicates p<0.05 compared to control)

	Drosophila melanogaster							*Drosophila Simulans*		
	Greece	Japan	F. Pol.	Florida	Gabon	Kenya	**All**	Greece	Kenya	**All**
	Synonymous Diversity x10^{-2} π_s									
Control	**0.79**	**0.95**	**1.19**	**1.19**	**1.34**	**1.55**	**1.47**	**2.03**	**2.62**	**2.79**
Immune	**0.89**	**1.03**	**1.25**	**1.26**	**1.45**	**1.60**	**1.56**	**1.97**	**2.46**	**2.62**
Humoral	0.80	1.01	1.17	1.19	1.43	1.53	1.50	2.07	2.62	2.79
Cellular	1.19	1.14	1.46	1.52	1.62	1.87	1.82	1.87	2.37	2.52
Melanisation	0.73	1.05	1.42	1.21	1.53	1.68	1.68	1.86	2.30	2.51
RNAi	0.39	0.49	0.70	0.61	0.66	0.80	0.71	0.90	1.02	1.07
Recognition (Cellular)	0.81	0.98	1.17	1.01	1.45	1.35	1.37	3.20	2.84	3.60
Recognition (Humoral)	1.22	1.25	1.63	1.55	1.53	1.78	1.71	1.85	2.27	2.47
Signalling	0.84	0.99	1.21	1.21	1.53	1.79	1.77	1.81	2.43	2.48
AMPs	0.64	1.38	1.03	1.34	1.75	1.30	1.35	2.28	2.84	2.94
	Non-synonymous Diversity x10^{-3} π_a									
Control	**0.89**	**1.03**	**1.27**	**1.21**	**1.51**	**1.63**	**1.78**	**1.53**	**2.06**	**2.20**
Immune	**1.31**	**1.32**	**1.56**	**1.69**	**1.96**	**1.95**	**1.94**	**1.77**	**2.35**	**2.48**
Humoral	1.36	1.30	1.62	1.73	2.04	2.14	2.09	1.87	2.34	2.54
Cellular	1.37	1.48	1.50	1.77	1.91	1.67	1.70	1.68	2.63	2.60
Melanisation	1.16	1.58	1.06	1.19	1.39	1.26	1.26	1.69	1.73	2.06
RNAi	0.50	0.75	1.21	1.22	1.66	1.46	1.56	1.41	2.09	1.92
Recognition (Cellular)	1.21	1.61	1.77	1.43	1.75	2.30	2.29	1.91	2.14	2.35
Recognition (Humoral)	1.82	2.12	2.23	2.50	2.75	2.32	2.37	2.42	3.70	3.70
Signalling	0.65	0.74	0.78	0.76	1.02	1.01	1.05	0.97	1.33	1.38
AMPs	1.87	2.22	2.32	3.42	3.04	3.85	3.69	3.42	3.85	4.13
