# Supplementary material for: Quantifying Adaptive Evolution in the Drosophila Immune System
Source: PLoS Genet. 2009 Oct 23;5(10):e1000698. doi: 10.1371/journal.pgen.1000698 (PMC2759075; doi:10.1371/journal.pgen.1000698)
Supplement: Text S1 — Detailed supplementary methods. (0.06 MB DOC) [file pgen.1000698.s030.doc]

**Supporting text 1: Detailed methods**

**Geographic sampling**

*Drosophila melanogaster* were sampled from six populations: Kenya (collected in Nairobi in 2005 by Jenny Carpenter), Gabon (N'Toumn and Franceville, 2002, Bill Ballard and Sylvain Charlat), French Polynesia (2005, Sylvain Charlat), Japan (Yamagata, 2006, Masayoshi Watada), Florida (Apshawa, 2005, Jenny Carpenter) and Greece (Athens, 2005, Natasa Fytrou). *D. simulans* were sampled from populations in Kenya (Nairobi, 2005, Jenny Carpenter) and Greece (Athens, 2005, Natasa Fytrou). The flies were kept as isofemale lines, and eight wild-type chromosomes were sampled from each population by crossing four pairs of lines and keeping a single female F1 fly from each cross for DNA extraction. This gives a total of 48 *D. melanogaster* chromosomes and 16 *D. simulans* chromosomes.

**Genes**

We sequenced a group of genes that have an immune function and a control group with no known immune function. We selected a subset of approximately 130 immune genes based either on direct experimental evidence of their immune function, or on their membership of an unambiguously immune-related family (e.g. anti-microbial peptides, turandots, TEPs). Each immune gene was matched by a control gene. The only criteria used to select these control genes were that they were ~100kb away from the immune gene and had no known immune function. Primers were designed from the *D. melanogaster* genome, with each target gene positioned near the centre of a 5kbp fragment. Where genes were substantially longer than 5kbp, multiple overlapping amplicons were used. This provided a large number of additional (occasionally partial) non-immune genes that happened to fall within the target fragments, and these were analyzed as control genes if they were represented by >100bp. A full list of genes, locations and categorizations is given in Table 1 of Supporting Text 1. Primer sequences are available from the authors.

**DNA extraction and PCR**

DNA was extracted using DNeasy kits (Qiagen) according to the manufacturer’s protocol. Each DNA extraction was amplified using GenomiPhi (GE Healthcare), which has an error rate approximately 100 times lower than *Taq* polymerase [1], and produced enough template DNA for PCR. In six of the populations, the DNA was extracted from each of the four flies separately, and the four DNA extractions from each population were quantified using SYBR green fluorescence, and then combined in an equimolar mixture. In the remaining two populations (*D. melanogaster* from Kenya and Japan), a single DNA extraction was performed on the four flies (the protocol was modified for the final two populations due to the difficulty of accurately quantifying the template in the samples processed first).

PCR (Qiagen, LongRange PCR) used to amply the regions outlined above. Where the PCR failed new primers were designed, but if these also failed, the locus was excluded. Similarly, where primers designed for *D. melanogaster* failed to work in *D. simulans*, one round of re-design was done using the *D. simulans* genome, but loci that failed again were excluded. This led to a smaller dataset for *D. simulans*. Note that this seems unlikely to strongly bias results, as most primers were positioned far outside the coding sequence of targeted genes.

**Sequencing and short-read mapping**

PCR products from each pooled population were quantified using the brightness of bands on an agarose gel and mixed in approximately equimolar amounts. To remove unincorporated nucleotides, primers and PCR product of the incorrect length, this mixture was then run on an agarose gel, the band excised, and the DNA purified from the gel slice. Pooled PCR products from each population were fragmented, ligated to adaptors, and sequenced using the Solexa-Illumina platform according to the manufacturer’s instructions. A separate lane of the Solexa-Illumina chip was used for each population, with repeats as necessary to give at least 5 million high-quality mapped reads per population.

The 36bp sequencing reads were aligned to the published *D. melanogaster* (r5.7) or *D. simulans* genome (r1.1) using MAQ [2], allowing for up to 2 mismatches between the read and the target sequence. For a small minority of targeted genes the genomic guide was unreliable (e.g. *Dscam*) or absent from the genome, and in these cases guide sequences for alignment were taken from Genbank. All reads with a quality score less than 20 (Q20) and a mapping score less than 10 were excluded, as were all reads that mapped to more than one location in the genome. Approximately 85-95% of reads mapped successfully to the genome, except in the *D. melanogaster* Athens population in which only 41% were mapped. This resulted in between 5.3M mapped reads and 15.8M mapped reads per population (Table 1, below), however there was no clear pattern with regard to which populations showed high or low mapping success. The variation is therefore likely to be due simply to experimental conditions, and in particular the low mapping success for the *D. melanogaster* Athens population may be due to poor fragmentation (see below).

Fragmentation of long PCR products can lead to extreme variation in coverage depth, and although the spatial distribution of relative read depths along the fragment is reported to be highly correlated between experiments[3], absolute values can vary substantially. We found that although the spatial distribution of relative read depth was similar between population samples and between loci - showing extreme peaks at the ends of PCR amplicons with low coverage circa 100bp from the ends (Figure S2) - the proportion of reads within the ‘peaks’ varied greatly. This distribution of read depths across PCR products is most likely due to uneven fragmentation of the 5kbp PCR products, and in particular, the low coverage and high peaks in the *D. melanogaster* Athens sample may reflect poor fragmentation of that sample (compare ‘Japan’ with ‘Athens’ in Figure S2).

Read depth can also vary according to mapping success. In particular MAQ only maps short reads that have a small number of mismatches to the reference sequence (up to 2 in this case), and consequently the mapping success and thus read-depth will be lower at sites where there is a mismatch with the reference sequence. This effect is detectable in our data, both in the downward bias of read depth at polymorphic sites relative to monomorphic sites (Figure S1; e.g. median depth 98 *vs.* 101 in Japan, and 263 *vs.* 289 in Florida) and also in a regression of read depth on the proportion of surrounding sites that are polymorphic (Figure S2). However, although this effect will cause us to miss some polymorphisms, most sites still have extremely high read depth and thus high power to detect even singleton polymorphisms (see “Data quality” below). Of potentially greater concern is that that mismatches by one allele (but not the other) may result a systematic upward bias in estimated frequency of the reference allele (which is also more likely to be the higher-frequency allele). However, this effect is not detectable in our data, as the intercept of a regression of minor allele frequency estimated using the short-read approach on that derived from Sanger sequencing is not significantly different from zero (Figure S3).

We also examined the effect of base composition on read depth. Firstly, sequences with a specific GC content may be easier to fragment, ligate, and/or sequence for biophysical or chemical reasons. Secondly, regions that differ systematically in GC content may also differ in the ease with which reads can be mapped. For example, in *Drosophila,* highly repetitive and non-coding regions tend to be AT-rich, and this could indirectly lead to a reduced mapping success. In our data there are highly significant (though relatively weak) effects of local GC content on read-depth. When all of the *D melanogaster* data are considered (including non-coding regions that were not analyzed), those populations with low coverage show a slight increase in read depth with increasing GC, while those with extremely high coverage show a decrease with increasing GC content (Figure S5). We hypothesize that this may reflect a qualitative difference in the success of mapping reads in repetitive AT-rich regions. When the analysis is limited to the coding regions, which are relatively GC-rich, there is a negative relationship between GC content and read depth in all populations (Figure S6). Because the strength of the effect varies considerably between populations that have similar read depths, this may reflect variation in the fragmentation process.

**Identifying polymorphic sites**

Following alignment using MAQ, the high quality (Q20 and above) base calls were tabulated by genomic position, and sites were recorded as polymorphic if read depth was greater than 20-fold at Q20 and the minor allele frequency was greater than 5% of the total. Assuming equal mixing of the template DNA, 20-fold coverage yields 73% power to detect an allele appearing only once in the 8 sampled chromosomes. Median coverage at polymorphic sites ranged between 98-fold (Japan, 99% power) and 263-fold (Florida, 99.99% power), and increasing the minimum coverage threshold for inclusion had no qualitative effect on our analysis (Figure S25).

Sites were assigned to coding or non-coding sequence using the GFF annotation provided by FlyBase for the corresponding genome release, and classified as synonymous or non-synonymous using the method of **Nei and Gojobori**[4] as implemented in the ‘SNAP’ PERL script [5].

**Data quality**

The need to accurately quantify template DNA, the possibility of allele-specific PCR amplification or failure (‘allelic dropout’), the opportunity for PCR-induced mutation, and the failure to map reads with more than 2 mismatches all have the potential to degrade data quality. To assess the potential impact of these effects, we re-sequenced 11 amplicons in the Athens (Greece) population samples of *D. melanogaster* and *D. simulans* using traditional Sanger sequencing (total length = 12415bp). These regions were amplified with different primers than were used for the Solexa-Illumina sequencing, allowing us to detect the effects of allelic dropout. Across this region we detected a total of 31 errors (polymorphic status miscalled: no site was miscalled as the wrong base), giving a per-site accuracy rate of 99.8%. Using these data we were able to quantify the rate of false positives (i.e. sites which appear polymorphic in the short-read dataset, but are not in the Sanger re-sequencing), false negatives (i.e. sites which appear monomorphic in the short-read data, but are actually polymorphic in the Sanger data), and to assess the quality of diversity estimates.

False positives may be introduced either by error in the Solexa-Illumina sequencing, or through mutations incorporated during the genomic amplification or Long-PCR. Such errors are likely to appear at low frequency, where they will have little impact upon pairwise diversity (π) but a potentially larger impact on Watterson’s θ and on the estimated number of synonymous and non-synonymous polymorphisms when all frequency classes are included. In particular, because non-synonymous sites are *ca.* 3-fold more common than synonymous sites, but true non-synonymous diversity is *ca.* 10-fold lower than synonymous diversity, such errors may dramatically inflate the count of non-synonymous polymorphisms. In our raw data, there is a large excess of T→C and A→G changes amongst variants with a minor allele frequency of <1% as compared to variants with a minor allele frequency of ≥5% (Figure S7), consistent with PCR-induced mutational patterns [6]. This suggests that PCR-induced false positives could indeed occur, although the error rates of polymerases are sufficiently low [6] that it is unlikely that the same PCR mutation will occur on multiple times at the same site. Therefore, to reduce the rate of false positives, we treated sites as polymorphic only if ≥5% of the high quality base-calls agreed on the minor allele. Of 177 putative polymorphic sites identified in the short-read dataset, 167 were also present in the Sanger sequences, suggesting that 94% of the variants identified were genuine polymorphisms. As a proportion, the rate of false positives was higher in *D. melanogaster* (91% correct) than *D. simulans* (96%), as would be expected from the lower diversity in *D. melanogaster*. The minor allele read-frequency of the false-positives was relatively low (8%) compared to the 5% minimum threshold for inclusion and the expected frequency of 12.5% for singletons. Calculated across all 6 *D. melanogaster* populations, this is a minor-read frequency of <1.3%. This means that none of these sites will appear in analyses which exclude low frequency variants (Figure S16) and therefore suggests that false positives had no impact on our primary results.

False negatives may be introduced either by allelic dropout during PCR, or when the read-frequency of the singleton polymorphisms (i.e. present in 1 chromosome of 8) falls below the required 5% minimum threshold for inclusion, or when mapping fails due to high divergence between the amplified allele and the reference genome. The effect of the 5% threshold alone is relatively small: assuming eight equally–represented template genotypes and our observed distribution of read-depths, this approach will only cause us to miss between 1.7% and 0.03% of true singleton polymorphisms (Athens and Florida read-depth distributions, respectively. Distributions are shown in Figure S1). Moreover, it has no appreciable impact upon our primary conclusions regarding the rate of adaptive evolution (Figure S16). Of greater concern is the effect of unequal amplification and allelic-dropout during PCR or mapping. Of 188 polymorphic sites identified in the Sanger data, 167 were also present in the short-read dataset, suggesting that we detect 89% of SNPs. However, most of the missing polymorphisms were due to allelic dropout at a single *D. simulans* locus, which can be traced to a segregating indel at a long-PCR primer-binding site (this suggests allelic dropout is likely to affect only a small proportion of loci: 1 in 22 tested). Excluding this locus, the overall power to detect polymorphic sites is 95%. Many of the missing SNPs occur at a low frequency (two-thirds were singletons or doubletons). Because the exclusion of segregating sites with a minor-allele frequency below 30% has little impact (Figure S16), it seems unlikely that a failure to detect these polymorphisms will impact upon our primary conclusions.

Uneven mixing of template DNA, uneven amplification by PCR and mapping failures will reduce the correlation between allele frequency in the sample and read-frequency in the short-read dataset, and lead to poor estimates of genetic diversity. Based on polymorphic sites present in both the short-read data and the Sanger sequenced loci, the correlation between minor allele frequency and read frequency is ρ = 0.71 (Figure S4). However, errors in estimates of the per-site allele frequency appear to have very little effect on estimates of genetic diversity (π or θ_w_) per locus, as these statistics calculated from Sanger data are very highly correlated with those estimated from short-read data (Pearson’s ρ = 0.94 and ρ = 0.90, respectively; Figure S8).

Table 1 in Supporting Text1: Read numbers and mapping

| **Sample** | **Raw Reads** | **Mapped reads** | **% mapped** |
| --- | --- | --- | --- |
| *D. melanogaster* |  |  |  |
| Japan | 5604967 | 5309768 | 94.70% |
| Kenya | 7189118 | 6631064 | 92.20% |
| Gabon | 8254665 | 6860353 | 83.10% |
| Athens | 16914592 | 7047107 | 41.70% |
| French Polynesia | 14094851 | 12190043 | 86.50% |
| Florida | 17445084 | 15862847 | 91% |
| *D. simulans* |  |  |  |
| Kenya | 8653757 | 6445041 | 74.50% |
| Athens | 10785487 | 9815085 | 91% |

**References cited in Supporting Text 1**

1. Esteban J, Salas M, Blanco L (1993) Fidelity of phi 29 DNA polymerase. Comparison between protein-primed initiation and DNA polymerization. J Biol Chem 268: 2719-2726.

2. Li H, Ruan J, Durbin R (2008) Mapping short DNA sequencing reads and calling variants using mapping quality scores. Genome Research 18: 1851-1858.

3. Harismendy O, Ng P, Strausberg R, Wang X, Stockwell T, et al. (2009) Evaluation of next generation sequencing platforms for population targeted sequencing studies. Genome Biology 10: R32.

4. Nei M, Gojobori T (1986) Simple Methods For Estimating The Numbers Of Synonymous And Nonsynonymous Nucleotide Substitutions. Molecular Biology And Evolution 3: 418-426.

5. Korber B (2000) HIV Signature and Sequence Variation Analysis. In: Rodrigo AG, Leam GH, editors. Computational Analysis of HIV Molecular Sequences. Dordrecht, Netherlands: Kluwer Academic Publishers. pp. 55-72.

6. Zylstra P, Rothenfluh HS, Weiller GF, Blanden RV, Steele EJ (1998) PCR amplification of murine immunoglobulin germline V genes: Strategies for minimization of recombination artefacts. Immunology and Cell Biology 76: 395-405.
